# Preoperative immune landscape predisposes adverse outcomes in hepatocellular carcinoma patients with liver transplantation

**DOI:** 10.1038/s41698-021-00167-2

**Published:** 2021-03-26

**Authors:** Sang-Ho Yoon, Seo-Won Choi, Suk Woo Nam, Kyoung Bun Lee, Jin-Wu Nam

**Affiliations:** 1 Department of Life Science, College of Natural Sciences, Hanyang University, Seoul, Republic of Korea; 2 Department of Pathology, College of Medicine, The Catholic University of Korea, Seoul, Republic of Korea; 3 Department of Pathology, College of Medicine, Seoul National University, Seoul, Republic of Korea; 4 Research Institute for Convergence of Basic Sciences, Hanyang University, Seoul, Republic of Korea; 5 Research Institute for Natural Sciences, Hanyang University, Seoul, Republic of Korea

**Keywords:** Cancer microenvironment, Hepatocellular carcinoma, Tumour biomarkers

## Abstract

Immune class in hepatocellular carcinoma (HCC) has been shown to possess immunogenic power; however, how preestablished immune landscapes in premalignant and early HCC stages impact the clinical outcomes of HCC patients remains unexplored. We sequenced bulk transcriptomes for 62 malignant tumor samples from a Korean HCC cohort in which 38 patients underwent total hepatectomy, as well as for 15 normal and 47 adjacent nontumor samples. Using in silico deconvolution of expression mixtures, 22 immune cell fractions for each sample were inferred, and validated with immune cell counting by immunohistochemistry. Cell type-specific immune signatures dynamically shifted from premalignant stages to the late HCC stage. Total hepatectomy patients displayed elevated immune infiltration and prolonged disease-free survival compared to the partial hepatectomy patients. However, patients who exhibited an infiltration of regulatory T cells (Tregs) during the pretransplantation period displayed a high risk of tumor relapse with suppressed immune responses, and pretreatment was a potential driver of Treg infiltration in the total hepatectomy group. Treg infiltration appeared to be independent of molecular classifications based on transcriptomic data. Our study provides not only comprehensive immune signatures in adjacent nontumor lesions and early malignant HCC stages but also clinical guidance for HCC patients who will undergo liver transplantation.

## Introduction

Hepatocellular carcinoma (HCC) is a leading cause of cancer-related death and is associated with a myriad of both intrinsic and extrinsic risk factors, including viral hepatitis^1,2^. Because viral infections (hepatitis B virus (HBV) or hepatitis C virus (HCV)) can drive chronic inflammation in the liver, HCC is reported to have a strong correlation with cirrhosis^3^. Although extensive inflammatory signals in premalignant disease can impact immune activity in later tumors, the detailed features of these inflammatory stages in terms of immune infiltration and dysregulation remain to be elucidated. For the treatment of HCC, surgical resection (partial hepatectomy (PH)) is the main option for the majority of cases; otherwise, liver transplantation (total hepatectomy (TH)) can be carried out, often improving survival rates^4^. As the incidence of transplantation has increased, tumor relapse after grafting has become an urgent issue. However, it is still uncertain how the preestablished immune context in the prehepatectomy period is associated with relapse in the posthepatectomy period^5,6^.

Cancer immunotherapy is a strategy for attacking tumor cells by stimulating the host’s own immune system or introducing engineered immune cells. Although many have witnessed marvelous successes with immune checkpoint inhibitors, this approach has not been widely applied in HCC^7^. However, a recent study identified an immune-specific class of HCC in a group of HCC patients^8^, suggesting that successful immunotherapy might be possible in HCC. In fact, nivolumab and pembrolizumab, anti-PD-1 checkpoint inhibitors, have already been approved by the U.S. Food and Drug Administration for patients with late-stage HCC treated with sorafenib^9,10^. Hence, to identify immunotherapy responders among HCC patients, a better understanding of the tumor microenvironment (TME) and the discovery of significant biomarkers in conjugation with operational methods are necessary.

Here, using computational deconvolution of bulk RNA sequencing (RNA-seq) data, we examined immune cell signatures of 124 samples, comprising various stages in tumor-adjacent nontumor and malignant tumors, from a Korean HCC cohort that had undergone liver transplantation and surgical resection. We then explored the relationships between immune cell contents and clinical outcomes to identify prognostic cell types in the TH group. We found that infiltration of regulatory T cells (Tregs) prior to transplantation was an independent predictor of a poor outcome. Furthermore, comprehensive analysis of prehepatectomy immune landscapes delineated how the immune cell network is dynamically reshaped during HCC development.

## Results

### Transcriptomic analysis of HCC and adjacent nontumor lesions

**Fig. 1:**
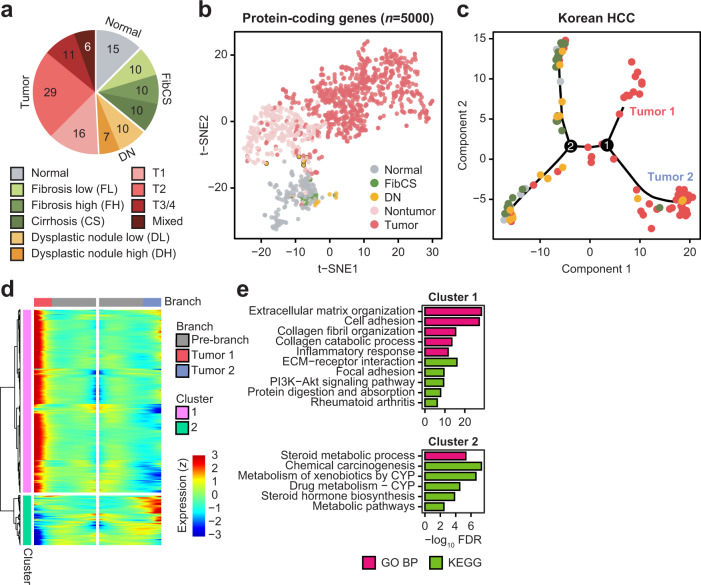
Overview of Korean HCC cohort characteristics. **a** Pie chart of the number of samples in each stage of histopathological classification. T1 ~ 3/4 = T of the TNM staging system; mixed = combined HCC and intrahepatic cholangiocarcinoma. **b** t-SNE analysis using the 5000 most highly expressed protein-coding genes in the meta-dataset (*n* = 1179). **c** Trajectory analysis of Korean HCC using Monocle 2. Colors are the same as in **b**. Numbers on the trajectory line indicate branching points. Two paths of tumor samples are noted as Tumors 1 and 2. **d** Branch expression analysis of two paths (Tumors 1 and 2) of branch 1 in **c**. **e** GO terms and KEGG pathways enriched in each cluster in **d**. The top-five significant terms (*FDR* < 0.05) were visualized.

We sequenced the transcriptomes of a total of 124 samples, comprising 62 malignant tumors, 47 adjacent nontumors, and 15 normal samples, from a Korean HCC cohort (Fig. 1a and Supplementary Table 1). Most of our tumor samples were sequenced in early stage or grade; about two-thirds of them were acquired from patients having TH. To explore the relationship between disease stages, samples were projected into two-dimensional space using principal component analysis (PCA) of the top 1000 most variable protein-coding genes (Supplementary Fig. 1a). Tumor samples were clearly separated from nontumor samples, whereas nontumor samples represented an intermediate state. In particular, dysplastic nodule (DN) samples were projected along with the tumor samples, whereas those from fibrotic and cirrhotic liver tissue were close to the normal samples (Supplementary Fig. 1a, inset). Because the adjacent nontumor samples were separated into two distinct states, they were categorized as either fibrosis low/high grade and cirrhosis (FibCS) or DN (DN low/high grade) (Fig. 1a, outer rim).

To compare these projection results with those of other cohorts, 925 RNA-seq samples from three publicly available HCC cohorts (TCGA-LIHC, *n* = 418; RIKEN, *n* = 447; GSE77509, *n* = 60)^11,12^ as well as a normal liver cohort from GTEx (*n* = 132)^13^ were projected together with ours using t-SNE^14^ after batch correction^15^. The normal, nontumor, and tumor samples were separately projected from each other (Fig. 1b and Supplementary Fig. 1b)^16^. FibCS samples were closely related to the GTEx and Korean nontumor samples, whereas the high-grade DN samples were related to the other nontumor samples, perhaps indicating shared features between developing cancer and tumor-adjacent tissue. In fact, an estimation of immune and stromal fractions^17^ showed that the DN and nontumor groups formed an intermediate state between the FibCS and tumor groups (Supplementary Fig. 1c). In addition, the HCC samples across the cohorts displayed an immune-depleted TME, although some samples showed immune- and stromal-enriched features. Because our HCC samples were aligned sequentially from various nontumor stages to tumors in the PCA and t-SNE plots, we applied a trajectory analysis using Monocle 2 to verify transitions in transcriptomic programs during disease progression (Fig. 1c). Using the normal samples as a root state, nontumor samples were first separated from tumors. These nontumor samples (state 5) differed from those in the root state (state 1) in the level of immune cell infiltration (Supplementary Fig. 1d). Tumor samples were enriched with proliferative programs and relatively depleted of immune signatures as depicted with ESTIMATE scores (Supplementary Fig. 1c, e, f). The samples were further divided into two branches and characterized by extracellular matrix interactions and inflammatory responses (Tumor 1) or metabolic pathways (Tumor 2) (Fig. 1c–e). Because the tumor samples were binarized based on inflammatory signatures, we further investigated the TME in detail.

### Estimation of immune infiltrates using deconvolution

**Fig. 2:**
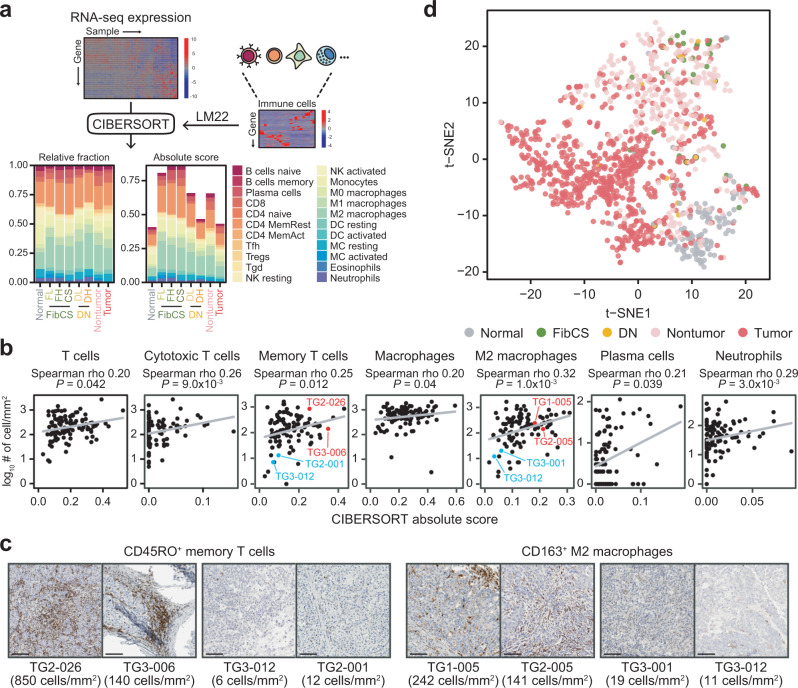
Deconvolution of immune cells and validation with IHC-derived cell counts. **a** Schematic workflow of deconvolution using CIBERSORT. **b** Spearman correlations between CIBERSORT absolute score (*X*-axis) and IHC cell count (*Y*-axis). **c** Representative samples highly infiltrated with or lacking (marked as red or blue in **b**, respectively) memory T cells or M2 macrophages in tumor tissue. The average number of cells are shown on a scale with units of cells/mm^2^. Scale bars: 100 µm. **d** t-SNE analysis of the integrated dataset based on CIBERSORT absolute scores. Samples are color-coded by disease stage.

Because a number of patients displayed an immune-enriched molecular signature, we investigated immune cell types that were populated in the TME and their associations with disease states. To address these questions, relative and absolute fractions of 22 immune cell types were inferred using CIBERSORT^18^, a computational method that predicts immune cell fractions using gene expression profiles (Fig. 2a). To validate the inferred fractions, immunohistochemistry (IHC)-based cell counts of seven major immune cell types (CD3^+^ total T cells, CD8^+^ cytotoxic T cells, CD45RO^+^ memory T cells, CD68^+^ macrophages, CD163^+^ M2 macrophages, MUM1^+^ plasma cells, and MPO^+^ neutrophils) were compared using the other specimens from same patients, and all of them correlated with the inferred absolute score (*P* < 0.05; Spearman’s correlation ≥0.2; Fig. 2b). Because a discrepancy between RNA and protein abundances has been repeatedly reported with a range from 0.35 to 0.5 across tissues^19–21^, the range of the correlation that we observed is thought to be fairly valid. For example, TG2-026 and TG3-006 correlated with high absolute scores for memory T cells, specifically showing 850 and 140 cells/mm^2^, respectively, whereas TG3-012 and TG2-001 correlated with low absolute scores and had only 6 and 12 cells/mm^2^, respectively (Fig. 2c, left). Similarly, TG1-005 and TG2-005 correlated with high absolute scores for M2 macrophages (242 and 141 cells/mm^2^, respectively), whereas only 19 and 11 cells/mm^2^ were observed in TG3-001 and TG3-012, which correlated with low absolute scores (Fig. 2c, right).

Based on the inferred absolute scores, samples from the Korean and other HCC cohorts were projected using PCA (Korean cohort; Supplementary Fig. 2a) and t-SNE (Korean + others; Fig. 2d and Supplementary Fig. 2b). Disease groups were separated from each other, but different cohorts were mixed together (Fig. 2d and Supplementary Fig. 2b). However, FibCS and DN samples were barely clustered and mixed with nontumor samples, indicating the heterogeneity and dysregulation of their immune composition compared to normal liver tissue. In short, inferred immune fractions could be used as a proxy for the immune landscape in our HCC cohort and identified distinguishing features in premalignant stages in terms of immune infiltration that were not observed in the previous analysis.

### Dynamics of immune infiltrations during HCC development

**Fig. 3:**
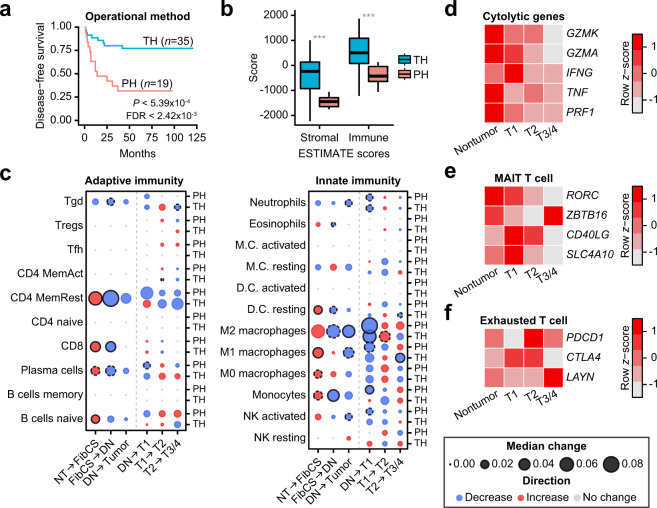
Landscape of changes in 22 major immune cell types during HCC development. **a** Kaplan–Meier DFS estimates for patients treated with each operational method (*n* = 54). **b** Comparison between stromal and immune scores of TH and PH. Welch’s *t*-test, ***P < 0.001. For each boxplot, the center line represents the median. The upper and lower limits of each box represent the 75th and 25th percentiles, respectively. The whiskers represent the highest and the lowest data points still within the 1.5× interquartile range. **c** Median fraction changes for 22 immune cell types in TH and PH in the Korean HCC cohort. Circle radii represent the magnitude of the change in median fractions between the two stages indicated below the box, and colors specify the direction of the change. Blue = decrease; red = increase; gray = no change during disease progression. Solid and dotted outlines indicate FDR and nominal *P* < 0.05, respectively (Welch’s *t*-test). **d–f** Changes in the expression of cytolytic genes (**d**), MAIT (**e**), or exhausted (**f**) T-cell markers during HCC development in TH. The scale of the changes was standardized as a *z*-score, which is shown in the scale bar.

Because the Korean cohort included 34 HCC patients plus one patient with a high-grade DN who underwent TH (*n* = 35 with survival information), whereas other cohorts did not, we next compared the TH patients to those who underwent PH (*n* = 19 with survival information) in terms of immune infiltration. First, the TH group showed better disease-free survival (DFS) rates (*P* < 5.39 × 10^−4^, log-rank test; Fig. 3a and Table 1), although the tumor stage and grade in the TH and PH groups were comparable to each other. Other than DFS, a few clinical parameters differed between TH and PH, probably representing patient selection or different clinical procedures prior to transplantation. For example, patients treated with embolization or ablation (pretreatment) were only found in the TH group who displayed elevated levels of fibrosis. Moreover, the tumors in the TH group showed higher immune and stromal contents and immune cell fractions than those in the PH group (Fig. 3b; stromal score, *P* = 1.62 × 10^−4^; immune score, *P* = 8.17 × 10^−4^, and Supplementary Fig. 2c; *P* = 9.08 × 10^−3^, Welch’s *t*-test).

**Table 1:** Comparison of clinical parameters between total and partial hepatectomy patients in the Korean HCC cohort.

Parameters	Group	TH	(*n* = 35)	PH	(*n* = 19)	*P* (Fisher’s exact test)
Survival	Dead	4	(11.43%)	2	(10.53%)	NS
	Alive	31	(88.57%)	17	(89.47%)	
Recur/meta	Yes	8	(22.86%)	13	(68.42%)	*1.51 × 10^−3^
	No	27	(77.14%)	6	(31.58%)	
Tumor stage	Stage I/II	29	(82.86%)	12	(63.16%)	NS
	Stage III+	6	(17.14%)	7	(36.84%)	
TNM (*T*)	T1/2	29	(82.86%)	14	(73.68%)	NS
	T3/4	6	(17.14%)	5	(26.32%)	
Grade	G1/2	31	(88.57%)	13	(68.42%)	NS
	G3	4	(11.43%)	6	(31.58%)	
Edmondson grade	ES1/2	24	(68.57%)	11	(57.89%)	NS
	ES3/4	10	(28.57%)	7	(36.84%)	
	NA	1	(2.88%)	1	(5.26%)	
Angioinvasion	Yes	9	(25.71%)	11	(57.89%)	**3.73 × 10^−3^
	No	26	(74.29%)	8	(42.11%)	
Viral infection	HBV	31	(88.57%)	8	(42.11%)	***1.21 × 10^−5^
	HCV	4	(11.43%)	2	(10.53%)	
	None	0		9	(47.37%)	
METAVIR activity	A0	0		2	(10.53%)	NS
	A1/2	5	(14.29%)	16	(84.21%)	
	NA	30	(85.71%)	1	(5.26%)	
METAVIR fibrosis	F1/2	0		10	(52.63%)	***5.84 × 10^−6^
	F3/4	33	(94.29%)	9	(47.37%)	
	NA	2	(5.71%)	0		
Pretreatment	Yes	21	(60.0%)	0		***5.06 × 10^−6^
	No	13	(37.14%)	18	(94.74%)	
	NA	1	(2.88%)	1	(5.26%)	
Child–Pugh	A	11	(31.43%)	18	(94.74%)	***6.93 × 10^−7^
	B/C	24	(68.57%)	0		
	NA	0		1	(5.26%)	
Family history	Yes	16	(45.71%)	7	(36.84%)	NS
	No	10	(28.57%)	10	(52.63%)	
	NA	9	(25.71%)	2	(10.53%)	
Parameters	(Units)	Mean ± SD		Mean ± SD		*P* (Welch’s *t*-test)
Age	(year)	52.71 ± 8.09		59.95 ± 9.87		**0.010
Weight	(kg)	67.50 ± 10.44		66.16 ± 8.09		NS
Height	(cm)	166.34 ± 7.38		164.93 ± 7.12		NS
AFP	(ng/ml)	41.22 ± 75.68		38.43 ± 51.71		NS
Tumor size	(cm)	3.25 ± 2.28		7.75 ± 5.27		***5.84 × 10^−6^

*P* values were calculated using the Fisher’s exact test or Welch’s *t*-test.

*NA* not applicable, *NS* not significant, *SD* standard deviation.

*P < 0.05; **P < 0.01; ***P < 0.001.

The Pre-Cancer Genome Atlas project highlighted the importance of investigating premalignant lesions to understand carcinogenesis^22^, and our tumor-adjacent nontumor samples offer an opportunity to unveil molecular/immune changes that occur during HCC development. To examine dynamic changes in immune cell contents, median fraction changes and correlations (Fig. 3c and Supplementary Fig. 3a, b) between 22 immune cell types were measured across disease stages. When nontumor samples from the Korean HCC cohort were considered, immune cells showed a sharp increase in FibCS, indicating elevated inflammatory responses and infiltration. However, most cell types were merely correlated with each other (Supplementary Fig. 3b, fibrosis/cirrhosis). In contrast, many of the cell types in DN samples were decreased (Fig. 3c), but significant associations with protective immunity were observed, including CD8^+^ T cells, plasma cells, and M1 macrophages (Supplementary Fig. 3b, DNs). These networks were maintained in early tumor stages but began to disappear in later stages; M2 macrophages started to establish strong correlations with other cell types. During tumor development, immune cell changes in PH samples were concordant across cohorts, with gradual depletion of the majority of cell types except for Tregs and macrophages. In TH samples, however, M1 and M2 macrophages increased in T1 to T2 but were depleted in later stages, whereas Tregs, NKs, and plasma cells were increased compared to PH samples (Fig. 3c). We also observed depletion of cytolytic activity (CYT) and mucosal-associated invariant T-cell markers^23,24^, and an increase in T-cell exhaustion over HCC progression, which indicates a process of immunosuppression in TH (Fig. 3d–f). We lastly compared inflammation between paired nontumor and tumor samples; however, there were no associations in either the ESTIMATE immune score or CIBERSORT absolute scores (Supplementary Fig. 3c)^25^.

### Tregs as a predictor of a high risk of relapse in TH

**Fig. 4:**
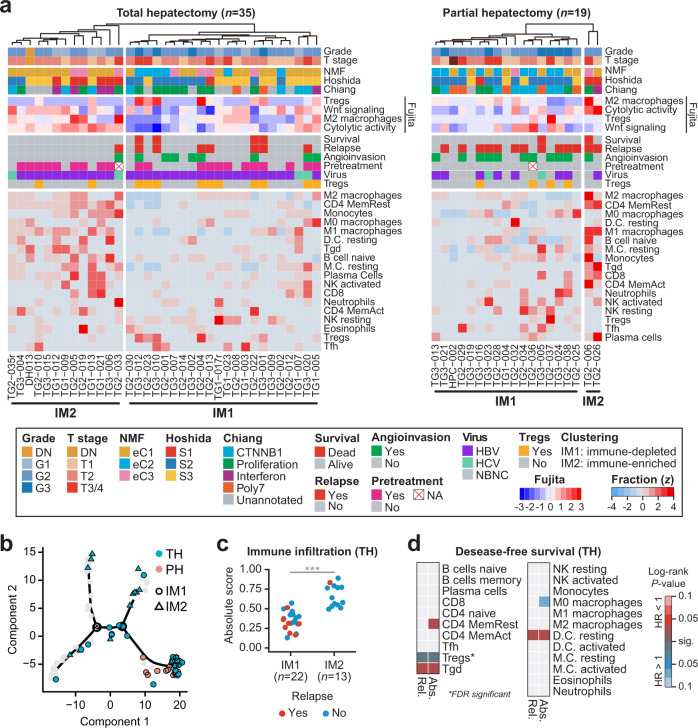
Identification of immune cell types associated with clinical outcomes in total hepatectomy. **a** Clustering of immune cell fractions in TH (*n* = 35; left) and PH (*n* = 19; right). Row dendrograms are not shown. Naive CD4 T cells and memory B cells in TH and eosinophils in PH were not inferred across all samples and were therefore excluded. Color codes are specified in the box. **b** Two immune clusters, IM1 and IM2, on the trajectory identified in Fig. 1c. Different colors and shapes indicate the operational method and immune cluster, respectively. **c** Comparison between absolute scores of the IM1 and IM2 clusters in TH. Welch’s *t*-test, ***P < 0.001. **d** Clinical associations of 22 immune cell types. Abs. absolute score, Rel. relative fraction. The bar color represents the significance of log-rank *P* values. When a high fraction of cells is correlated with a poor outcome, the appropriate box is colored blue (lower half). When a high fraction of cells is correlated with a good outcome, the box is colored red (upper half). *Significant after FDR adjustment.

Although patients with similar tumor stage and grade who underwent TH showed a better clinical outcome than those who underwent PH, some displayed recurrence and/or metastasis (Fig. 3a and Table 1). Moreover, some clinically important immune cell types, such as M1/M2 macrophages or Tregs, were differentially infiltrated between TH and PH that may contribute to disease relapse (Fig. 3c). To explore this issue, we clustered Korean HCC samples based on immune cell contents (Fig. 4a). The TH group was clearly separated into two clusters (IM1 and IM2) that differed in immune infiltration and with respect to other clinical features such as survival. The immune-depleted TH samples in the IM1 cluster displayed lower amounts of immune cell infiltration (*P* < 5.41 × 10^−7^; Welch’s *t*-test, Fig. 4c), higher amounts of Wnt/β-catenin signaling pathway activity and immunosuppressive cell types (Fujita subclass)^25^, depletion of the Hoshida S1 subtype (*P* = 2.15 × 10^−3^, One-sided Barnard’s test; 5.87 × 10^−3^, Fisher’s exact test), and a marginally increased relapse rate (*P* = 0.084) (Fig. 4a). PH samples were also segregated by immune infiltration, which showed that the majority of samples were clustered into the immune-depleted IM1 cluster (Fig. 4a). These immune clusters were also segregated by PCA using CIBERSORT (Supplementary Fig. 2d) and tumor branches in the trajectory analysis (Fig. 4b and Supplementary Fig. 4b). We clustered Korean HCC samples based on gene expression using non-negative matrix factorization (NMF)^26^, but we were unable to obtain clearly distinguished immune clusters with this method (Fig. 4a and Supplementary Fig. 4a).

Adjacent nontumor samples (*n* = 47) were stratified into four groups using immune cell scores (Supplementary Fig. 5a, b, PL-IM1 ~ 4). These samples were largely separated into two states in the trajectory analysis (Fig. 1c and Supplementary Fig. 1d, states 1 and 5) and state 5, which showed a higher load of immune infiltration is preferentially found in PL-IM3 and 4. In contrast, immune-depleted state 1 is prevalent in PL-IM1 and 2. These nontumor immune clusters were independent of those defined in paired tumor samples. Macrophages, which were the most abundant cell type in fibrosis/cirrhosis samples, showed dynamic changes during HCC development (Fig. 3c and Supplementary Fig. 3b). Two types of differentiated macrophages with opposing functions, M1 and M2, were enriched in PL-IM3 and PL-IM2, respectively, although these two immune clusters are mainly associated to a single NMF cluster, PL2 (Supplementary Fig. 5a, c). Likewise, gene expression-based clustering again failed to separate immune-enriched from immune-depleted samples (Supplementary Fig. 5a, d, NMF PL1 ~ 3). Overall, our immune cell clustering, in contrast to the expression-based clustering, was able to identify elevated immune signatures that could be ignored by other types of molecular clustering.

We next sought to identify specific immune cell types correlated with clinical outcomes. Of the 22 immune cell types analyzed, infiltration of Tregs was found to be a strong indicator of tumor relapse in TH after false discovery rate (FDR) correction (*FDR* < 6.39 × 10^−3^ for the absolute score; *FDR* < 1.62 × 10^−3^ for the relative fraction; Fig. 4d) and in multivariate analysis (*P* < 0.039; Supplementary Fig. 6a). The number of FOXP3^+^ cells determined by IHC also showed a positive correlation with the absolute fraction of Tregs (*P* = 0.043; Supplementary Fig. 6b, g). Tregs are known to be the main source of immune exhaustion and are linked to poor outcomes in human cancers, including HCC^27–31^. As expected, patients with Treg infiltration displayed immunodepletion (Fig. 4a and Supplementary Figs. 4c and 6c). Moreover, Tregs were confined in the tumor, inversely correlated with the amount of total T cells, and were associated with the expression of a chemokine receptor, *CCR8* (Supplementary Fig. 6d–f)^32,33^. In the PH group, we found that activated NK cells and M2 macrophages, which were previously reported to be localized in tumor-surrounding stroma^34,35^, were correlated with poor outcome (Supplementary Fig. 4d). To investigate whether our nontumor samples could better represent macrophage infiltration in paratumor tissues, we stratified paired nontumor samples by immune cell fractions and tested the relationship with prognosis. In summary, we found that M2 macrophage and activated NK cell infiltration predicted poor DFS rates in PH patients using adjacent nontumor samples; predictions could be further improved by considering tumor pairs together, but in a small number of patients (Supplementary Fig. 5e). Taken together, our data suggest that established Treg infiltration prior to liver transplantation predicts poor outcomes in the TH group.

### Associations of Treg infiltration with tumor metastasis signals and pretreatment

**Fig. 5:**
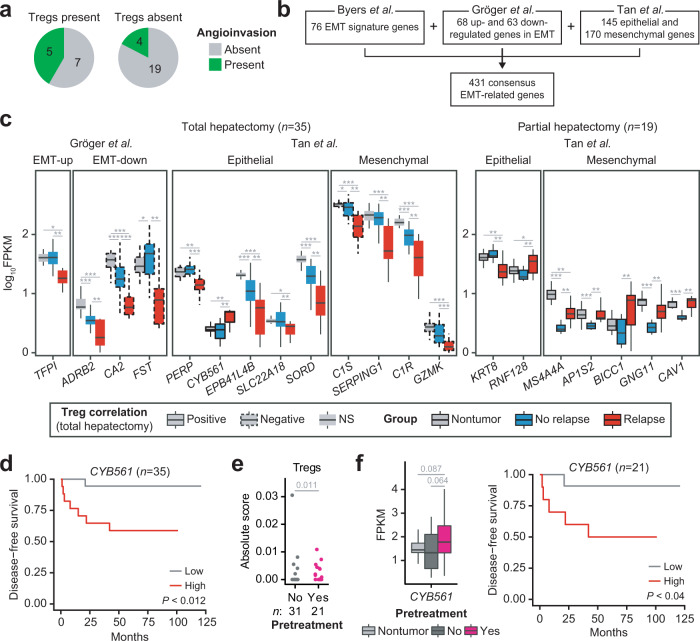
Clinical relevance of pretreatment in the total hepatectomy group. **a** Angioinvasion and Treg infiltration in the TH group. **b** 431 EMT markers pooled from three independent studies. **c** Dysregulated EMT marker expression upon tumor relapse in the TH (left, *n* = 35) and PH (right, *n* = 19) groups. The correlation with Tregs was examined only in the TH group. Epithelial = highly expressed in epithelial cells, mesenchymal = highly expressed in mesenchymal cells, EMT-up/down = up-/down-regulated expression, respectively, during EMT. Each group is indicated as a colored box (gray for nontumor, blue for no relapse, and red for relapse), and the Treg correlation of the TH group is indicated by the outline (solid for positive, dotted for negative, and no line for no correlation). **d** Kaplan–Meier survival analysis for patients stratified by high and low *CYB561* expression in the TH group. **e** Absolute score of Tregs upon pretreatment in the Korean HCC cohort (*n* = 52). Two samples with missing pretreatment information were excluded. *P* value was estimated using the One-sided Barnard’s test. **f** Dysregulation of *CYB561* expression upon pretreatment and Kaplan–Meier survival analysis of the pretreatment subgroup. *P* values (left) were estimated using the Welch’s *t*-test. For boxplots, the center line represents the median. The upper and lower limits of each box represent the 75th and 25th percentiles, respectively. The whiskers represent the highest and the lowest data points still within the 1.5× interquartile range.

In addition to the high risk of relapse, patients with Treg infiltration displayed a marginally higher proportion of angioinvasion than those without Tregs (Fig. 5a; *P* = 0.073; One-sided Barnard’s test). Angioinvasion, a critical process for metastasis, can be induced by Tregs. In line with this point, Tregs have also been reported to promote metastasis of HBV^+^ HCC^36–38^. Hence, we searched for epithelial–mesenchymal transition (EMT) markers that were dysregulated. A total of 431 EMT-related genes were manually curated from independent studies (Fig. 5b)^39–41^. Thirteen and seven of these genes were found to be dysregulated in TH and PH samples, respectively (Fig. 5c). For example, among four dysregulated mesenchymal genes in TH (*C1S*, *SERPING1*, *C1R*, and *GZMK)*, three of them are associated with the complement cascade, and *GZMK* is an effector molecule of cytotoxic cells, suggesting suppression of immune responses. Six out of 13 dysregulated genes in the TH group were associated with Treg fractions, but only *CYB561* showed a positive correlation with the level of Tregs and tumor relapse (Fig. 5c, left, and d).

**Fig. 6:**
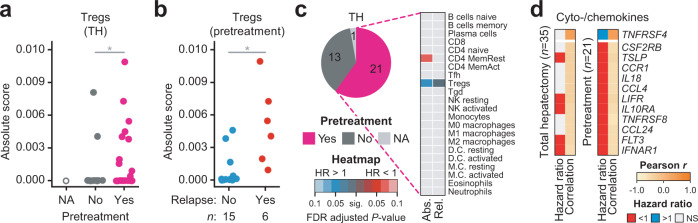
Association of pretreatment with the infiltration of Tregs in the total hepatectomy group. **a** Absolute score of Tregs upon pretreatment in TH. One-sided Barnard’s test, *P < 0.05. **b** Relapse status and absolute score of Tregs. Welch’s *t*-test, *P < 0.05. **c** Clinical associations of 22 immune cell types; Abs. absolute score, Rel. relative fraction. Colored boxes adjacent to specific cell types represent the significance of FDR-adjusted log-rank *P* values. The heat map is colored in the same manner as that used in Fig. 4c. **d** Correlations with Tregs and clinical associations of cytokines/chemokine ligands and receptors. The boxes, representing HRs, are colored in blue or red to indicate when relatively high expression was correlated with a poor or good outcome, respectively.

When other clinical backgrounds were examined in TH, infiltration of Tregs was not only associated with pretreatment in both Korean HCC and TH patients (*P* = 0.036, One-sided Barnard’s test; Figs. 5e and 6a) but was also found to be the sole predictor of tumor relapse in the pretreatment subgroup (*n* = 21, *FDR* < 0.056) (Fig. 6b, c). Intriguingly, *CYB561* expression was also upregulated upon pretreatment, and this gene was confirmed as a predictive biomarker in the pretreatment subgroup (Fig. 5f). Our results underscore the possible connection of pretreatment with the infiltration of Tregs and suggest that the expression of the epithelial marker *CYB561* can be used as a biomarker for Treg infiltration. In fact, considering additional Korean HCC cohorts (*n* = 701; TH = 320 and PH = 381) with clinical information, the relapse of TH patients was significantly biased to distant metastasis or recurrence with metastasis rather than local recurrence, compared to PH patients (*P* = 8.81 × 10^−9^, chi-square test; Supplementary Fig. 7a). Combined analysis with the TCGA cohort showed a similar trend as that of the extended Korean cohort (*P* = 1.98 × 10^−11^, chi-square test; Supplementary Fig. 7b). Furthermore, TH patients undergoing pretreatment appeared to have more relapses or metastasis than those without pretreatment (*P* = 3.04 × 10^−3^; *P* = 8.81 × 10^−3^, respectively, chi-square test; Supplementary Fig. 7c, d). These patients were neither biased to higher T stage nor Edmondson–Steiner grade (*P* = 0.29; *P* = 0.47, respectively, chi-square test; Supplementary Fig. 7e, f), indicating that the results were independent of patient selection.

### *TNFRSF4* is associated with infiltrating Tregs in the TH group

To further elucidate the molecular signatures related to the infiltration of Tregs, ligands and receptors for cytokines and chemokines in the Kyoto Encyclopedia of Genes and Genomes (KEGG) database were further examined. The expression of most cytokine/chemokine genes was inversely correlated with the Treg level (Fig. 6d). For example, the expression of *LIFR*, a metastasis suppressor in HCC^42^, was associated with improved survival rates and inversely correlated with Treg infiltration (*r* = −0.40 and −0.50 in the TH and pretreatment subgroups, respectively). Only *TNFRSF4* expression, which is known to be expressed in immunosuppressive Tregs^43,44^, was correlated with the Treg level (*r* = 0.60) and displayed an increased hazard ratio in the pretreatment subgroup (Fig. 6d), although its significance was marginal in the TH group (*P* < 0.075; Supplementary Fig. 8a, b). Expression of the gene encoding the TNFRSF4 ligand, *TNFSF4*, also showed an adverse impact on DFS; however, it was not associated with any of the cell types (Supplementary Fig. 8b).

## Discussion

Unlike other HCC cohorts, the HCC cohort we studied here included a number of patients who underwent liver transplantation in addition to those who underwent surgical resection. The profiling of immune landscapes showed that TH patients comprised two groups displaying distinct immune signatures that were strongly associated with the clinical parameters and outcomes of the patients. Generally, patients waiting for a transplant undergo pretreatment regimens (embolization or ablation)^45,46^. Although pretreatment could affect patient immune responses or even gene expression at the molecular level, comprehensive studies to understand these effects are unprecedented to our knowledge. In fact, patients undergoing pretreatment tended to display elevated Treg infiltration. Our study also showed that Treg infiltration was strongly associated with adverse signatures, such as angioinvasion or tumor relapse in TH. It is likely that Treg infiltration encourages early dissemination of tumor cells not detected pretransplantation. In fact, compared to relapsed PH patients, relapsed TH patients tended to have distant metastasis rather than local recurrence. In addition, Treg infiltration and pretreatment were both associated with expression levels of the *CYB561* gene, which could be utilized as a surrogate for the prediction of adverse outcomes in TH patients. Intriguingly, we noticed a positive correlation between the expression of *CYB561* and that of the hypoxia-inducible factor gene *HIF1A* in our TH patients and the TCGA cohort (Supplementary Fig. 6h), suggesting that *CYB561* expression might be associated with a hypoxic condition in tumors. Development of such a biomarker would be clinically meaningful because IHC or cell sorting is not always feasible and is often laborious, especially for studying a large number of patients.

Although the immune landscape shaped during tumorigenesis can affect the characteristics of cancer, how immune networks are established in the premalignant and malignant stages is unknown. Because our dataset also included pairs of tumor and nontumor samples in various stages, investigation of the immune network in this context offers an unprecedented snapshot of immune dysregulation during HCC development. Fujita and his colleagues performed transcriptomic and genomic analyses of samples from Japanese liver cancer patients and classified 234 samples into the following immune clusters: CYT, Treg, tumor-associated macrophage (TAM), or CTNNB1^25^. They reported that the inflammation in nontumor samples was not correlated with that of the paired tumor samples. Similarly, we found that immune features of normal/nontumor samples were uncorrelated with those of their tumor counterparts, although an inflammation signal was highly induced in fibrotic and cirrhotic samples (Supplementary Fig. 3c). In addition, our study found that inflammation surged in the early premalignant stages spanning from fibrosis to cirrhosis, then decreased to a level similar to that in the tumor samples (Supplementary Fig. 1c).

Although our analysis could recapitulate the immune network dysregulation, the nontumor samples in our study were not time serial but were rather from adjacent stroma in HCC patients; hence, immune profiles could be affected by nearby tumor lesions. However, because invasive sampling of premalignant tissue in cancer-free individuals is infeasible and because these diseases are already on the course of tumor development, our data provide a unique opportunity to investigate diverse aspects of premalignancy in the liver at high resolution. In addition, the significance of M2 macrophages in PH samples increased when we stratified samples based on fractions inferred from both tumor and paired adjacent nontumor samples. Activated NK cell signals followed similar trends with slightly higher *P* values. M2 macrophages, also known as TAMs, have been reported to support progression of tumors including HCC^35,47^; however, the significance of this cell type was ambiguous in previous studies using digital dissections^31,48–50^. This result could be partly due to the localization of the M2 macrophages, because they are frequently enriched in tumor-surrounding stroma, the site from which our nontumor samples were obtained^34,35^. Given these points, we anticipate that our preliminary results will highlight the importance of M2 macrophages and that the nontumor samples used in this study will be a supplementary resource for evaluating the immune features of the HCC microenvironment.

Deconvolution using gene expression profiles is a useful strategy for associating functions of immune or stromal compartments in bulk mixture samples. One caveat of using this strategy is that the performance could be heavily dependent on the quality and representativity of the reference matrix. In fact, CIBEROSRT and other methods use a reference matrix based on gene expression profiles from peripheral blood cells. Expression profiles in tumor tissues may differ substantially from that of normal cells and this variation may not be fully covered by current reference matrices. In this study, we correlated inferred CIBERSORT scores with IHC cell counts from the same patients, resulting in correlations ranging from 0.2 to 0.32. These significant yet low correlations could be partly caused by inconsistencies between RNA expression and protein abundance, by the representativity of the reference matrix used, or by tumor heterogeneity. Perhaps a reference matrix derived from HCC single-cell RNA-seq data or augmented with noncoding RNA expression profiles would enhance the correlation.

Our research identifies adverse effects of Treg infiltration in patients in the TH group who have undergone pretreatment. Moreover, we also argue that adjacent nontumor samples are valuable resources for monitoring the dysregulation of the immune network during HCC development. Based on our study, patient TME-based therapeutic approaches that modulate a specific immune cell type, combined with surgery or transplantation, would improve HCC patient survival rates.

## Methods

### Collection of samples

HCC and nontumor samples were collected from HCC or chronic liver disease patients, respectively, who had undergone surgical resection at Seoul National University Hospital between 2004 and 2009. A total of 62 tumor samples were collected, 25 of which had a paired sample—one from nontumor tissue and 24 from premalignant lesions. Of the 15 normal samples, one was collected from a metastatic cancer patient who underwent PH, and the other 14 were collected from patients with cholangiocarcinoma or polycystic liver disease after histological confirmation. Of the 47 nontumor lesion samples that we prepared, 24 were paired with tumor samples as mentioned above. Tissue samples were immediately snap-frozen and stored in liquid nitrogen. Then, matched formalin-fixed, paraffin-embedded blocks were made for histological diagnosis and immunohistochemical staining. In total, 124 samples from 98 patients were collected.

### Ethics approval and consent to participate

All tissue samples were obtained after receiving written informed consent from the patients according to the Declaration of Helsinki. This study was approved by the Institutional Review Board of Seoul National University Hospital (H-1501-042-639).

### High-throughput RNA sequencing (RNA-seq)

Liver tissues were removed and flash-frozen on dry ice, and RNA was harvested using Trizol reagent. One microgram of total RNA was extracted from samples and subsequently subjected to quantitative real-time PCR. RNA-seq libraries were constructed using an Illumina TruSeq RNA Sample Prep Kit (FC-122-1001, Illumina, CA). RNA-seq libraries were sequenced in paired-end reads using the HiSeq 2000 platform (LAS, Gimpo, South Korea).

Public RNA-seq data (*n* = 1057) were downloaded from the Genomic Data Commons portal (TCGA-LIHC, *n* = 418), European Genome-Phenome Archive (EGA; EGAD00001001880) (RIKEN, *n* = 447), National Center for Biotechnology Information (NCBI) Gene Expression Omnibus (GEO; GSE77509, *n* = 60), and database of Genotypes and Phenotypes (dbGaP; GTEx-liver, *n* = 132). Encrypted cip files (RIKEN) downloaded from the EGA were converted to fastq using an EGA client (version 2.2.4) with a provided decryption key. SRA files (GSE77509 and GTEx-liver) downloaded from NCBI GEO and dbGaP were converted to fastq using *fastq-dump* with a *--split-files* option in SRA Toolkit (version 2.8.0)^51^.

### Clinical data generation

Clinical information for 98 HCC patients, such as postoperative tumor recurrence or metastasis, survival, and preoperative treatment, was collected from existing medical records. Of the 54 patients with clinical information available, 35 underwent TH, and the remaining 19 underwent PH. Criteria for the determination of pathologic T stage followed the liver tumor, intrahepatic bile duct tumor, or perihilar bile duct tumor staging guidelines established by the American Joint Committee on Cancer^52^. Pathological information, such as angioinvasion, tumor differentiation, fibrosis grade, HCC grade, and DN status, was obtained from pathological reports and by slide review. Angioinvasion was defined as tumor thrombi in vascular lumen, which could be identified under microscopic examination of hematoxylin and eosin-stained glass slides.

### Preprocessing and analysis of RNA-seq data

For preprocessing, a total of 124 Korean RNA-seq data samples were first examined for sequencing quality using FastQC (version 0.11.5)^53^. Then, they were aligned to the human reference genome hg19 using Bowtie (version 1.0.0)^54^ with default parameters. Mismatch rates across aligned reads were calculated from the resulting bam files using an in-house Python script, and reads having a mismatch ratio higher than 10% in either end position were trimmed using Seqtk (version 1.0-r31)^55^. The remaining reads were then filtered using Sickle (version 1.33)^56^ if their Phred base quality score was lower than 20. The filtered RNA-seq reads were aligned to the human genome (hg19) using STAR aligner (version 2.5.2b)^57^ with a transcriptome annotation file (GENCODE version 19, GTF formatted) specified by a *--sjdbGTFfile* parameter. Read quantification was performed using featureCounts^58^ from the Subread package (version 1.5.1)^59^ with *-p*, -*t* *exon* and -*g* *gene_id* parameters with transcriptome annotation used in the alignment step. Two samples with low mapping rates (TG3-022 and TG1-025) were excluded from subsequent analyses, and the remaining 122 samples were used throughout this study. The resulting output files from featureCounts were aggregated and converted to fragments per kilobase of exon per million fragments mapped (FPKM) values using an in-house Python script. The FPKM values were subsequently normalized using quantile normalization to adjust intersample variations in R using the *normalize.quantiles()* function in the preprocessCore package^60^. Protein-coding genes (*gene_type “protein_coding”* in GTF) were then selected from the normalized expression table, and redundant genes were filtered out. The remaining number of protein-coding genes was 20,242. For tumor samples, 53 out of 62 had available clinical information (34 TH and 19 PH patients), and an additional DN high-grade sample with complete clinical data was included in the TH group.

For the other four HCC and normal liver tissue datasets (TCGA, RIKEN, GSE77509 HCC, and GTEx), only Sickle filtering was performed on the raw RNA-seq reads after a quality check using FastQC. Following transcriptome alignment, read quantification and normalization steps were performed with the protocol used with the Korean HCC cohort.

### Principal component analysis (PCA)

In total, 20,242 protein-coding genes were first sorted by expression variances across samples, and the 1000 most varying genes were selected. Using either these 1000 genes or CIBERSORT absolute scores for 22 immune cell types, PCA was performed in R using the FactoMineR package with default parameters. A total of five principal components (PCs) were extracted during the analysis even though only PC1 and PC2 are visualized in the figures.

### Batch correction and dimensionality reduction analysis of a meta-dataset

Before performing t-distributed stochastic neighbor embedding (t-SNE) analysis of the meta-dataset (Korean, TCGA, RIKEN, and GSE77509 HCC and GTEx-liver samples; *n* = 1179), normalized FPKM expression tables of the top 5000 highly expressed (sorted by median expression) protein-coding genes were merged. To diagnose batch effects potentially introduced by merging independent datasets, BatchQC^61^ was used in R. As expected, the batch effect prevailed in the meta-dataset and showed a higher level of explained variation (median = 20.11, 1st quartile = 10.19 and 3rd quartile = 32.87) than the “conditions” of a sample (four conditions were manually specified: nontumor, premalignancy (FibCS and DN), tumor, and normal liver (Korean Nontumor and GTEx)) (median = 9.287, 1st quartile = 4.681 and 3rd quartile = 15.48). To resolve the batch effect, batch adjustment using empirical Bayes (ComBat)^15^ was performed in R, and the adjusted expression table was subsequently subjected to another round of BatchQC. The batch effect after adjustment was significantly diminished to a median percent explained variation of 1.734 (1st quartile = 0.8105 and 3rd quartile = 3.326), whereas those of the conditions were only slightly affected (median = 6.047, 1st quartile = 2.512, and 3rd quartile = 11.8), indicating proper normalization of the batch effect. Negative expression values in the adjusted expression table were set to 0 during adjustment.

Then, t-SNE analysis was performed on the batch-corrected expression table using the Rtsne package^62^ with *dims* *=* *3* and *pca_scale* *=* *TRUE* or default parameters (*initial_dims* *=* *50*, *perplexity* *=* *30*, and *theta* *=* *0.5*) in R. The analysis was repeated using the top 500, 1000, 2000, and 5000 highly expressed genes; only the results for the top 5000 genes are shown in Supplementary Fig. 1b because the results all exhibit similar trends.

### Running ESTIMATE and the calculation of purity scores

Stromal, immune, and ESTIMATE scores were inferred using the ESTIMATE package (version 1.0.13) in R. ESTIMATE is an enrichment test evaluating the amounts of stromal or immune cells in a given microarray or RNA-seq sample using 141 stromal and 141 immune signature genes, and this test is performed in two steps. First, *filterCommonGenes()* was run with an *id* *=* *“GeneSymbol”* option to select the subset of genes to be used in enrichment tests from an input expression matrix. In total, 10,205 genes out of the 20,242 protein-coding genes in our expression table matched in the ESTIMATE test and were used for the enrichment test. Next, a function *estimateScore()* with *platform* *=* *“illumina”* was run to calculate stromal, immune, and ESTIMATE scores. A total of 138 stromal and 141 immune signature genes were found in our expression matrix. The calculated stromal, immune, and ESTIMATE scores were saved in a text file for later use. Because the purity score is not automatically converted from the ESTIMATE score when the *platform* *=* *“illumina”* option is specified in the *estimateScore()* function, an in-house Python script performed the task. The scores generated by ESTIMATE are summarized in Supplementary Table 3.

### Monocle 2 trajectory analysis

Read count matrix of Korean HCC samples were applied to Monocle 2 (http://cole-trapnell-lab.github.io/monocle-release/) for the trajectory analysis with default parameters. The 2127 highly variable genes were selected with criteria of *dispersion_empirical* *≥* 2 * dispersion_fit & mean_expression *≥* *10*. For each branching point, we used BEAM for branch expression analyses. Only genes significantly dysregulated (*Q* value < 0.00005) along each branch were used.

### Estimation of immune cell contents

The expression table for each dataset was separately uploaded to the CIBERSORT website (https://cibersort.stanford.edu) and run with the following CIBERSORT options: run relative and absolute modes together (beta), a LM22 reference file, 500 permutations, and disable quantile normalization. The resulting CIBERSORT absolute scores and relative fractions for the 22 immune cell types in our cohort are shown in Supplementary Table 4.

### IHC staining and interpretation

Quantification of immune cells (T cells, cytotoxic T cells, macrophages, plasma cells, and neutrophils) was assessed by IHC staining on tissue microarrays and automatic quantification by QuPath^63^. CD45Ro (Ventana; 790-2930; Mouse monoclonal (UCHL-1)) for total immune cells, CD3 (Ventana; 790-4341; Rabbit monoclonal (2GV6)) for total T cells, CD8 (NOVO; PA0183; Mouse monoclonal (4B11)) for cytotoxic T cells, Foxp3 (Abcam; ab20034; Mouse monoclonal (236A/E7)) for Tregs, CD68 (DAKO; M0814; Mouse monoclonal (KP1)) for macrophages, CD163 (NOVO; NCL-CD163; Mouse monoclonal (10D6)) for M2 macrophages, MUM1 (DAKO; M7259; Mouse monoclonal (MUM1P)) for plasma cells, and MPO (DAKO; A0398; Rabbit polyclonal) for neutrophils were used as cell type markers. Four-μm-thick glass slides were stained using Ventana BenchMark XT and OptiView universal DAB staining kit (Ventana #760-700). Staining procedure of automatic stainer is antigen retrieval (100 °C for 24 min in citrate buffer), peroxidase inhibition (37 °C for 4 min in 3% H_2_O_2_), primary antibody (37 °C for 16 min), Linker (HQ linker; 37 °C for 8 min), polymer amplification (HRP multimer; 37 °C for 8 min), chromogen by DAB (37 °C for 8 min), counterstaining by hematoxylin (37 °C for 8 min), and post counterstain (37 °C for 4 min).

### Validation of CIBERSORT immune cell types

For validation of the immune cell fractions inferred by CIBERSORT, IHC-based cell counts of eight cell types were performed with samples from matched patients (Supplementary Table 5). The immune cell types measured were as follows: CD3^+^ total T cells, CD8^+^ cytotoxic T cells, CD45RO^+^ memory T cells, FOXP3^+^ Tregs, CD68^+^ macrophages, CD163^+^ M2-type macrophages, MUM1^+^ plasma cells, MPO^+^ neutrophils, and CD45^+^ total leukocytes. To group cell types in the IHC results into categories such as “memory T cells” or “macrophages” using the CIBERSORT results, the following CIBERSORT cell types were aggregated: CD3^+^ total T cells = CD8 + CD4 naive + CD4 memory resting + CD4 memory activated + follicular helper + gamma-delta T cells; CD45RO^+^ memory T cells = CD4 memory resting + CD4 memory-activated T cells; CD68^+^ macrophages = monocytes + M0 macrophages + M1 macrophages + M2 macrophages. The IHC-based cell counts and CIBERSORT scores were correlated using the *cor.test()* function with *method* *=* *“spearman”* in R.

### t-SNE analysis using CIBERSORT absolute scores

Inferred CIBERSORT absolute fractions of 22 immune cell types in the Korean, TCGA, RIKEN, and GSE77509 HCC and GTEx-liver data were manually merged into a single file, and then dimension reduction analysis (t-SNE) was performed using the Rtsne R package. The specified parameters were *dims* *=* *3* and *pca_scale* *=* *TRUE* or the default settings. Batch correction was not performed, and a batch effect was not present in the t-SNE result.

### NMF signature genes and GO analysis

To cluster molecular subtypes in the Korean HCC cohort, a machine-learning approach, NMF (version 0.20.6), was performed in R. Before NMF, genes with a median expression level <1 FPKM were excluded using an in-house Python script. The NMF parameter *r* was set from 2 to 10, and the number of iterations per *r* (*nrun*) used a default (100 iterations) option. Then, the best performing *r* value was chosen by comparing cophenetic (reproducibility of a model), dispersion (stability of a model), evar (explained variance achieved by a model), and silhouette values. Signature genes in each NMF class were extracted with an in-house R script using the following NMF function: *basis(extractFeatures(<NMF object>, method* *=* *“kim,” format* *=* *“subset“))*.

For each HCC subtype, gene ontology (GO) analysis was performed with the list of signature genes via DAVID Bioinformatics Resources 6.8^64,65^. The “Functional Annotation Clustering” result in the “Combined View for Selected Annotation” section was downloaded for the interpretation of a subtype. For the visualization of GO analyses in Supplementary Figs. 4a and 5d, only the first significant GO term (FDR < 0.05) in the GO categories of GOTERM_BP_DIRECT, GOTERM_CC_DIRECT, GOTERM_MF_DIRECT, UP_KEYWORDS, and KEGG_PATHWAY was selected as a representative description of the cluster if multiple clusters were present with several statistically significant GO terms. When only one cluster was identified and harbored several significant GO terms (FDR < 0.05), all of the GO terms were visualized.

### Immune cell–cell network

The absolute scores of 22 immune cell types inferred by CIBERSORT in the Korean, TCGA, and RIKEN HCC cohorts were separated by annotated disease stages (nontumor; FibCS; DN low-/high-grade; and tumor T1-3/4 stages). Within a single stage, Pearson’s correlation between immune cell types was performed, and only cell–cell interactions with significant coefficients (*P* < 0.05) remained. The resulting correlation tables were visualized using cell fractions, correlation coefficients and *P* values in Cytoscape (version 3.5.1). In Cytoscape, absolute scores are presented by different font sizes, correlation coefficients by width and edge color (red = positive, blue = negative correlation coefficient) and correlation *P* values by the transparency of the edges. Legends for color and font size were generated using the more advanced version 3.8.2.

### Clustering analysis

For clustering of immune cell fractions, the “*heatmap.2*” function was specified as the hclust agglomeration method of “*complete*” and distance function of “*euclidean*” in R. For clustering of gene expression values, NMF was performed on expression matrices using the NMF package (version 0.20.6)^66^ with default parameters in R.

### Statistics

All statistical analyses were performed using R (version 3.2.3 or 3.5.3). Welch’s *t*-test and Spearman/Pearson correlations were performed to compare expression values or scores and relationships between groups, respectively. Enrichment of a class in a subtype or comparisons of binary classes were analyzed by the Barnard’s test. Survival analysis was performed using the survival R package (version 2.41-3)^67^. *P* values were adjusted for multiple-hypothesis testing using the function “*p.adjust()*” with the “*fdr*” method.

### Reporting summary

Further information on research design is available in the Nature Research Reporting Summary linked to this article.

## Supplementary information

Supplementary Material

REPORTING SUMMARY

## Data Availability

The data generated and analyzed during this study are described in the following data record: 10.6084/m9.figshare.13853033^68^. The normalized gene expression and raw data supporting the conclusions of this article are available in the NCBI Gene Expression Omnibus (GEO) database under accession number https://identifiers.org/geo:GSE148355^69^. Raw Immunohistochemistry data underlying Fig. 3 and Supplementary Fig. 6 are available in figshare at 10.6084/m9.figshare.13729081.v1^70^. A spreadsheet containing the 430 pooled genes related to the EMT process is available in figshare at 10.6084/m9.figshare.13729108.v1^71^. Clinical metadata for the extended Korean HCC cohorts, liver transplantation (LT), and surgical resection (non-LT) data are available in figshare at 10.6084/m9.figshare.13883321.v1^72^.

## Code availability

In-house R and Python codes that were implemented in this study are available on GitHub (https://github.com/sangho1130/KOR_HCC). Analyses were performed using default parameters, otherwise, described in “Methods.” All analyses were performed using Python (version 2.7.5), R (version 3.2.3 or 3.5.3), and R Studio (version 1.1.383). Detailed software versions are described in “Methods.”
