# Supplementary material for: Preoperative immune landscape predisposes adverse outcomes in hepatocellular carcinoma patients with liver transplantation
Source: NPJ Precis Oncol. 2021 Mar 26;5:27. doi: 10.1038/s41698-021-00167-2 (PMC7997876; doi:10.1038/s41698-021-00167-2)
Supplement: Supplementary file 1 — Supplementary Material [file 41698_2021_167_MOESM1_ESM.pdf]

SUPPLEMENTARY FIGURES

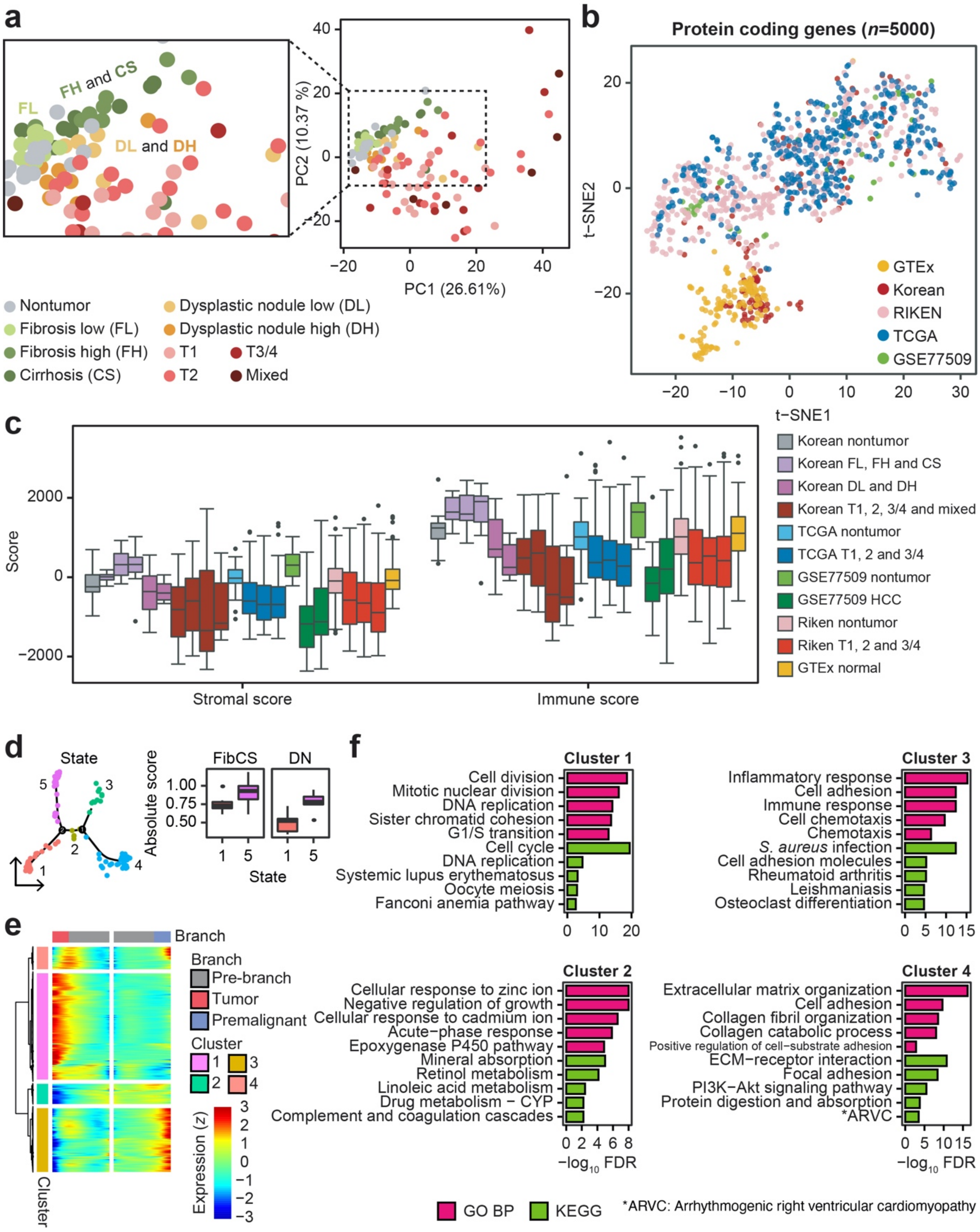

Supplementary Fig. 1. Transcriptomic dissections of an HCC and adjacent nontumor meta-dataset by disease stage and by cohort. a PCA projection of Korean HCC samples. The inset shows nontumor samples in the projection. **b** t-SNE

analyses using the 5,000 most highly expressed protein-coding genes in the meta-dataset (*n* = 1179), which included Korean, TCGA, RIKEN, GSE77509, and GTEx liver cohorts. The color of each point indicates the origin. **c** Stromal and immune scores of the meta-dataset inferred using ESTIMATE. **d** Comparison between CIBERSORT absolute scores of adjacent nontumor samples in two states of trajectory. **e** Branch expression analysis of nontumor and tumor paths of branch 2 in **Fig. 1c**. **f** GO terms and KEGG pathways enriched in the four clusters in (**e**). The top 5 significant terms (FDR-adjusted $P < 0.05$) were visualized. For boxplots, the center line represents the median. The upper and lower limits of each box represent the 75th and 25th percentiles, respectively. The whiskers represent the highest and the lowest data points still within the 1.5x inter-quartile range.

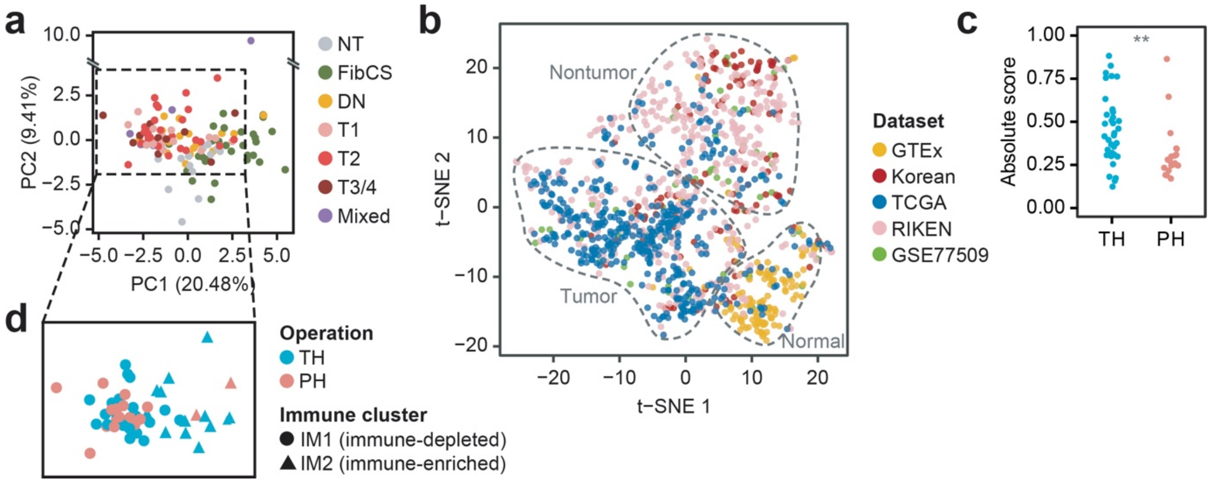

Supplementary Fig. 2. Immuno-genomic dissections of the Korean cohort and meta-dataset. a PCA result for the Korean samples based on CIBERSORT absolute scores of 22 immune cell types. The color of each point indicates the disease stage. **b** t-SNE analysis of the meta-dataset based on CIBERSORT absolute scores. Samples are colored by cohort. **c** Comparison between CIBERSORT absolute scores of TH and PH. Welch's t-test, **: $P < 0.01$. **d** The inset in (**a**). The color of each point indicates the type of surgical intervention and the shape indicates the immune cluster.

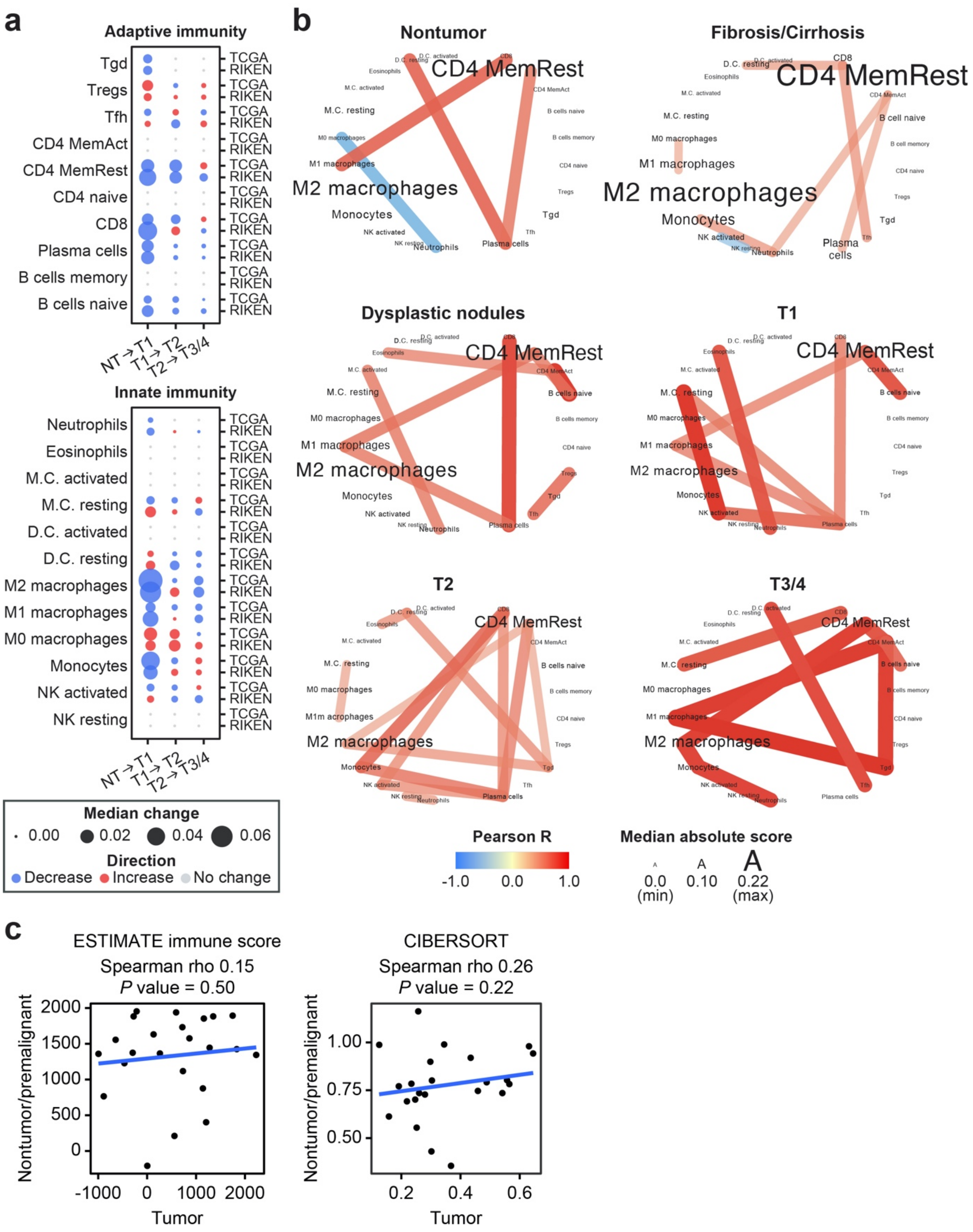

Supplementary Fig. 3. Immune cell landscape during the development of HCC. **a** Median fraction changes for 22 immune cell types in the TCGA and RIKEN HCC cohorts. Circle radii represent the magnitude of the change in median fractions between the two stages indicated below the box, and colors specify the direction of

the change. Blue = decrease; red = increase; gray = no change during disease progression. **b** Dynamic changes in the immune cell-cell network during HCC development in the Korean cohort. The font size is relative to the fraction of the corresponding type of immune cell. A red line specifies a positive correlation between two immune cell types, whereas a blue line specifies a negative correlation. The width and transparency of the lines denote the correlation coefficient and *P*-value, respectively. **c** Spearman correlations between ESTIMATE immune scores or CIBERSORT absolute scores of paired tumor (X-axis) and normal/nontumor (Y-axis) samples.

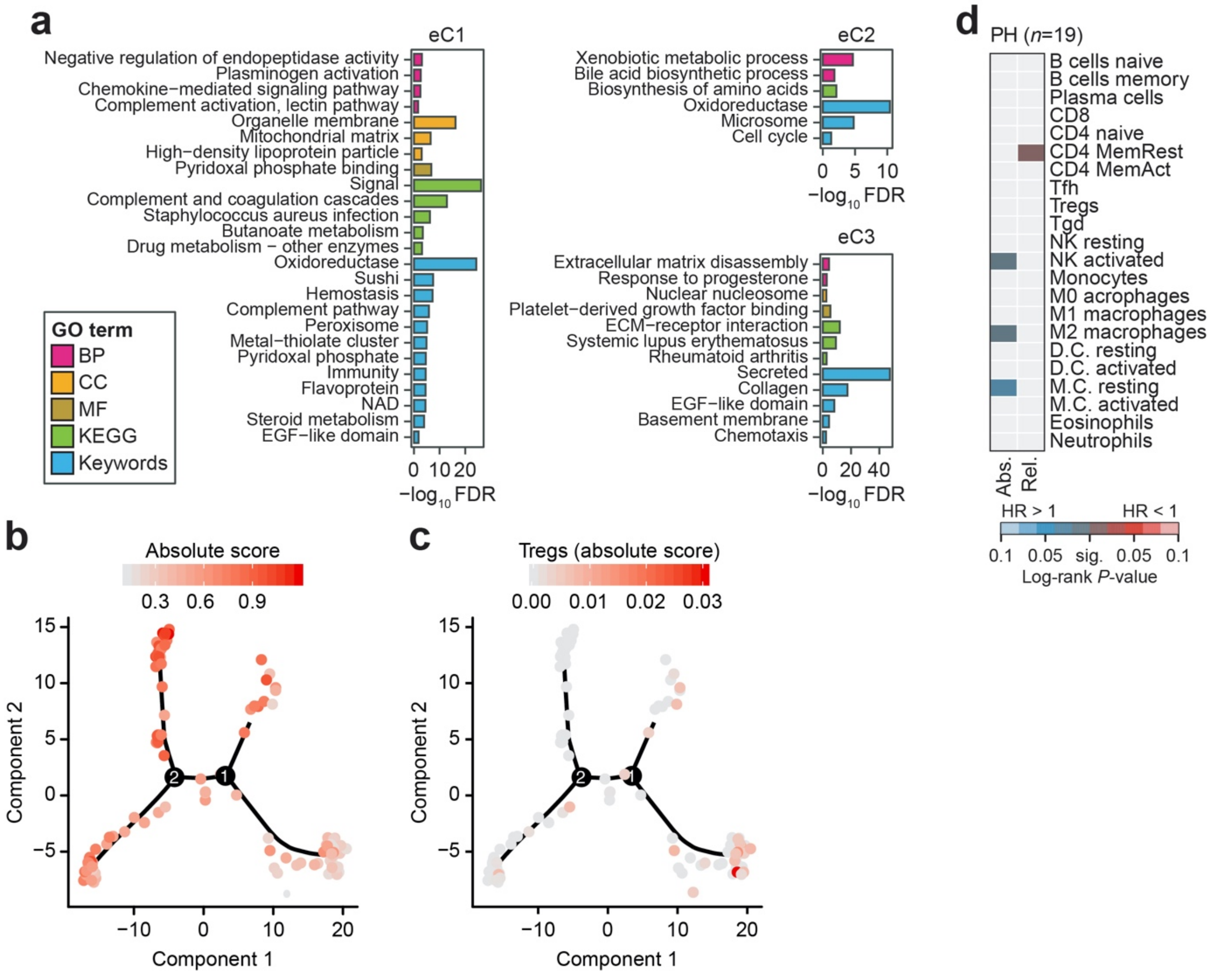

Supplementary Fig. 4. Hierarchical clustering of HCC samples by immune cell fraction. a Three expression-based molecular clusters (eC1, eC2, and eC3) found in the tumor samples (*n* = 54) using NMF. Representative GO terms from GO clusters are shown with FDR values on the -$\log_{10}$ scale. BP: biological processes; CC: cellular localization; MF: molecular functions; KEGG: KEGG pathways; and Keywords: UniProt knowledgebase keywords. **b** CIBERSORT absolute scores on the trajectory. **c** CIBERSORT absolute scores of Tregs on the trajectory. **d** Clinical associations of 22 immune cell types. Abs. = absolute score; Rel. = relative fraction. The bar color represents the significance of log-rank *P*-values. When a high fraction of cells is correlated with a poor outcome, the appropriate box is colored blue (left half). When a

high fraction of cells is correlated with a good outcome, the box is colored red (right half).

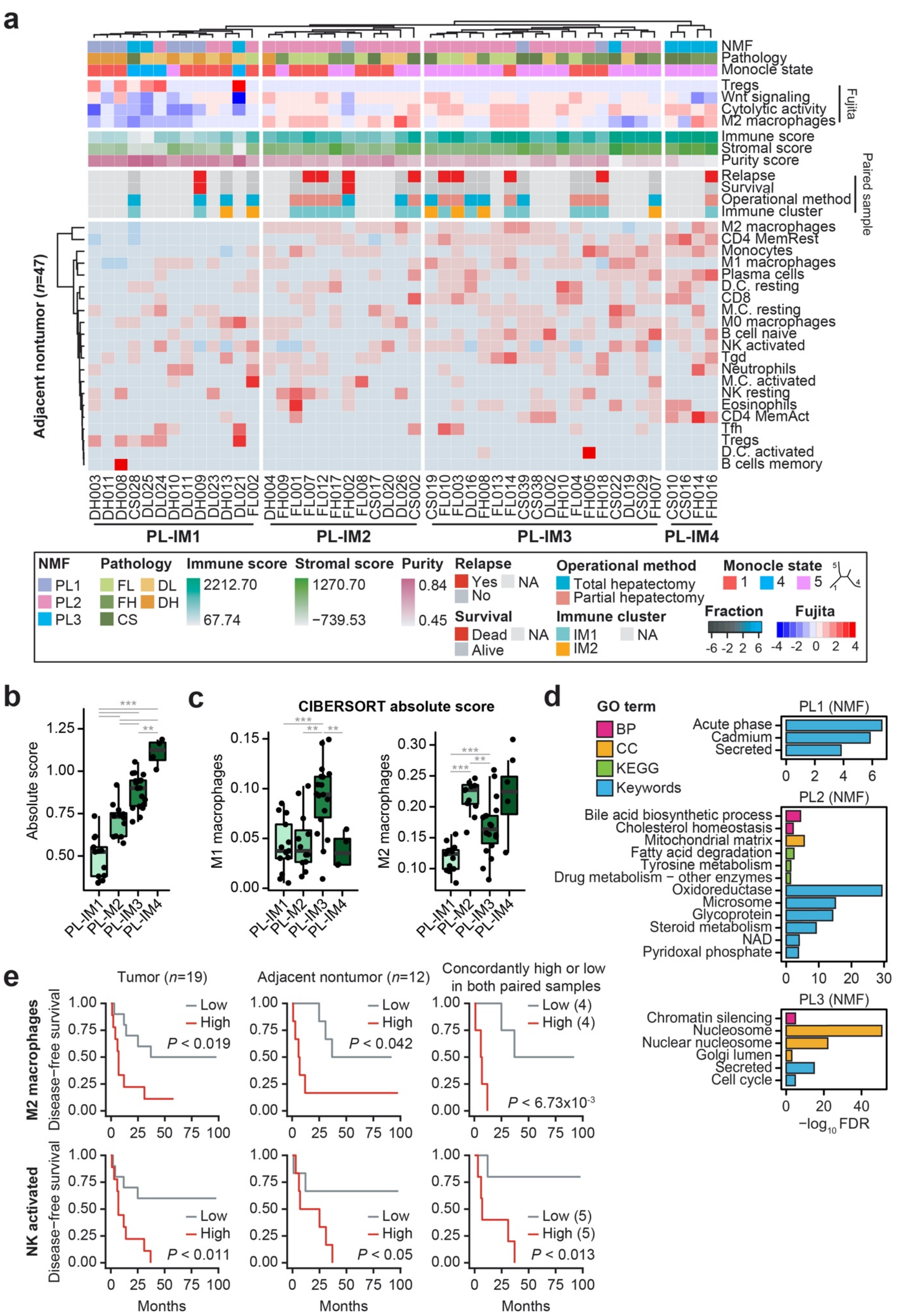
a
NMF
Pathology
Monocle state
Tregs
Wnt signaling
Cytolytic activity
M2 macrophages
Fujita
Immune score
Stromal score
Purity score
Relapse
Survival
Operational method
Immune cluster
Paired sample
Adjacent nontumor (n=47)
M2 macrophages
CD4 MemRest
Monocytes
M1 macrophages
Plasma cells
D.C. resting
CD8
M.C. resting
M0 macrophages
B cell naive
NK activated
Tgd
Neutrophils
M.C. activated
NK resting
Eosinophils
CD4 MemAct
Tfh
Tregs
D.C. activated
B cells memory
DH003 DH011 DH008 CS028 DL025 DL024 DH010 DL011 DH009 DL023 DH013 DL021 FL002
DH004 FH009 FL001 FL007 FL012 FH017 FH002 FL008 CS017 DL020 DL026 CS002
CS019 FL010 FL003 DL016 FH008 FL013 FL014 CS039 CS038 DL002 FH010 FL004 FH005 FH018 CS022 DL019 CS029 FH007
CS010 CS016 FH014 FH016
PL-IM1
PL-IM2
PL-IM3
PL-IM4
NMF
PL1
PL2
PL3
Pathology
FL
DL
FH
DH
CS
Immune score
2212.70
67.74
Stromal score
1270.70
−739.53
Purity
0.84
0.45
Relapse
Yes
NA
No
Survival
Dead
NA
Alive
Operational method
Total hepatectomy
Partial hepatectomy
Immune cluster
IM1
NA
IM2
Monocle state
1
4
5
Fraction
-6 -2 2 6
Fujita
-4 -2 0 2 4
b
Absolute score
1.25
1.00
0.75
0.50
***
**
PL-IM1
PL-IM2
PL-IM3
PL-IM4
c
CIBERSORT absolute score
M1 macrophages
0.15
0.10
0.05
0.00
***
**
**
M2 macrophages
0.30
0.25
0.20
0.15
0.10
***
***
**
PL-IM1
PL-IM2
PL-IM3
PL-IM4
d
GO term
BP
CC
KEGG
Keywords
PL1 (NMF)
Acute phase
Cadmium
Secreted
0 2 4 6
PL2 (NMF)
Bile acid biosynthetic process
Cholesterol homeostasis
Mitochondrial matrix
Fatty acid degradation
Tyrosine metabolism
Drug metabolism − other enzymes
Oxidoreductase
Microsome
Glycoprotein
Steroid metabolism
NAD
Pyridoxal phosphate
0 10 20 30
PL3 (NMF)
Chromatin silencing
Nucleosome
Nuclear nucleosome
Golgi lumen
Secreted
Cell cycle
0 20 40
−log10 FDR
e
Tumor (n=19)
Adjacent nontumor (n=12)
Concordantly high or low
in both paired samples
M2 macrophages
Disease−free survival
Low
High
P < 0.019
P < 0.042
Low (4)
High (4)
P < 6.73x10-3
NK activated
Disease−free survival
P < 0.011
P < 0.05
Low (5)
High (5)
P < 0.013
1.00
0.75
0.50
0.25
0.00
0 25 50 75 100
Months

Supplementary Fig. 5. Hierarchical clustering of 47 adjacent nontumor samples by immune cell fraction. a Clustering of immune cell fractions in adjacent nontumor samples (n = 47). Naive CD4 T cells were not inferred across all samples and were therefore excluded. Relapse, survival, operational method, and immune cluster data are from paired tumor samples. Color codes are specified in the box. **b** Comparison between CIBERSORT absolute scores of adjacent nontumor immune clusters PL-IM1~4. Welch's t-test, **: $P < 0.01$; ***: $P < 0.001$. **c** Comparison between CIBERSORT absolute scores of M1 (left) and M2 (right) macrophages of adjacent nontumor immune clusters PL-IM1~4. Welch's t-test, **: $P < 0.01$; ***: $P < 0.001$. **d** Three expression-based molecular clusters (PL1, PL2, and PL3) found in the adjacent nontumor samples using NMF. Representative GO terms from GO clusters are shown with FDR values on the $\log_{10}$ scale. BP: biological processes; CC: cellular localization; MF: molecular functions; KEGG: KEGG pathways; and Keywords: UniProt knowledgebase keywords. **e** Kaplan-Meier DFS analyses of patients stratified by M2 macrophage (top) or activated NK cell (bottom) fractions using tumor (n = 19, left), adjacent nontumor (n = 12, middle), and both tumor and adjacent nontumor samples (right). For boxplots, the center line represents the median. The upper and lower limits of each box represent the 75th and 25th percentiles, respectively. The whiskers represent the highest and the lowest data points still within the 1.5x inter-quartile range.

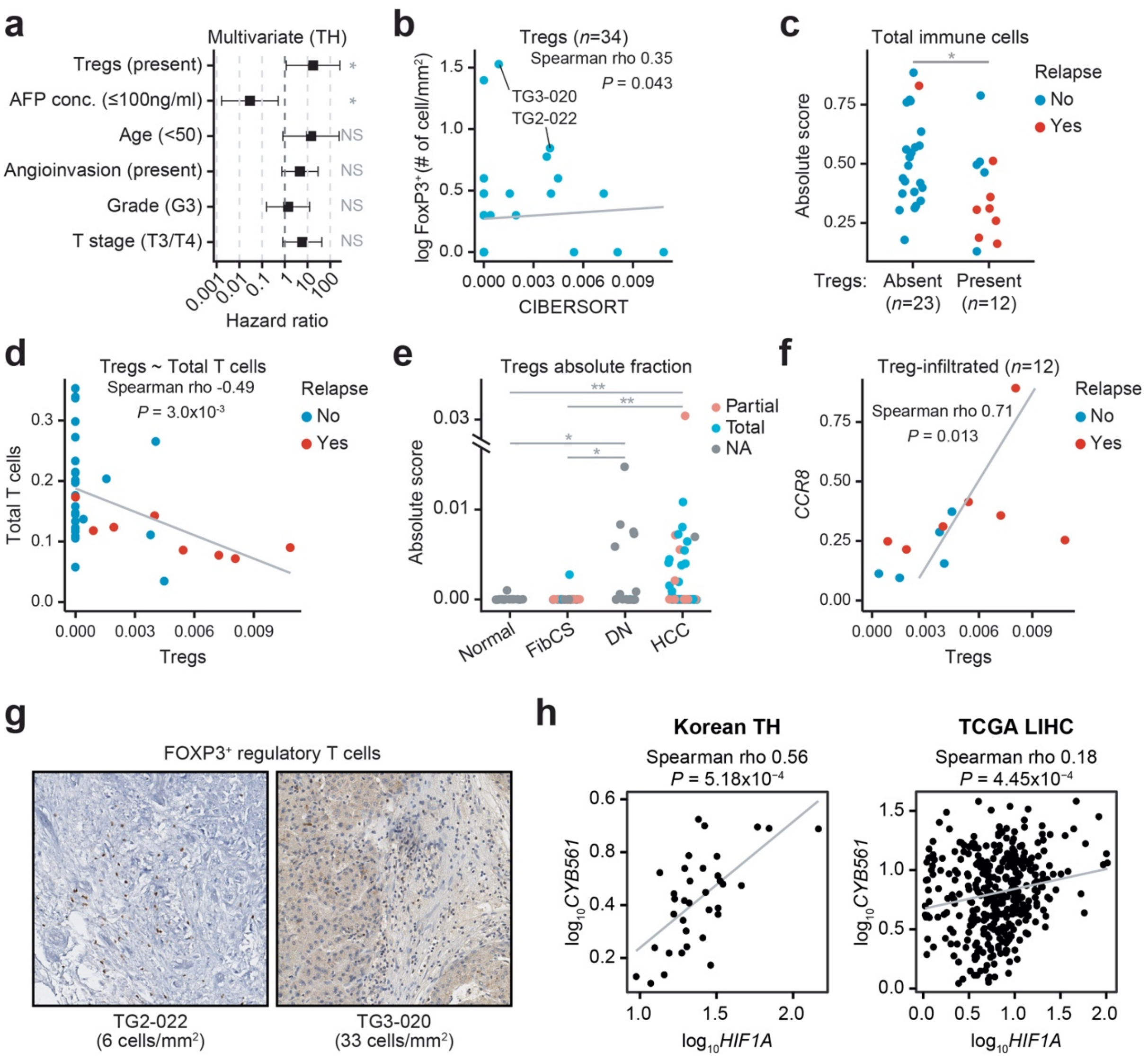

Supplementary Fig. 6. Characterization of infiltrating Tregs in the Korean total hepatectomy group. a Cox-proportional multivariate analysis of Tregs. *: $P < 0.05$ and NS = not significant. The error bars indicate the 95% confidence interval. **b** Spearman correlation between the CIBERSORT absolute scores and the IHC-derived cell count of Tregs in the TH group. **c** Decrease in total immune cell infiltration measured by absolute scores upon Treg infiltration. Whether tumor relapse occurred is indicated by the color code. Welch's t-test, *: $P < 0.05$. **d** Spearman correlation between Treg and total T cell fractions. The total T cell fraction was calculated by aggregation of the following CIBERSORT T cell types: CD8, CD4 naive, CD4 memory

resting, CD4 memory activated, follicular helper, and gamma delta T cells. **e** Treg fractions by disease stage. Surgical interventions (PH or TH) are color-coded. Surgical interventions in nontumor samples follow the corresponding intervention for the paired tumor sample. **f** Spearman correlation between the fraction of Tregs and *CCR8* gene expression. **g** Tumor tissue samples highly infiltrated with Tregs. Average numbers of cells are indicated under the samples with units of cells/mm^2. **h** Spearman correlation between the expression of *CYB561* and *HIF1A* in samples from Korean TH and TCGA cohorts.

a

Korean extended	TH (n = 35 + 320)	PH (n = 19 + 381)
Local recurrence	20	166
Metastasis	56	95

b

Korean extended with TCGA LIHC	TH (n = 355 + 1*)	PH (n = 400 + 368)
Local recurrence	20	287
Metastasis	56	141

c

Korean extended TH	w/ pretreatment (n = 21 + 177)	w/o pretreatment (n = 13 + 106)
Tumor-free	139	99
Relapse	51	14

d

Korean extended TH	w/ pretreatment (n = 21 + 177)	w/o pretreatment (n = 13 + 106)
Tumor-free	139	99
Metastasis	37	10

e

Korean extended TH	w/ pretreatment (n = 21 + 177)	w/o pretreatment (n = 13 + 106)
T3/4	28	12
T1/2	170	107

f

Korean extended TH	w/ pretreatment (n = 21 + 177)	w/o pretreatment (n = 13 + 106)
ES3/4	49	34
E1/2	148	85

Supplementary Fig. 7. Metastasis bias in total hepatectomy patients. a-b Contingency tables of recurrence or metastasis by operational method in extended Korean HCC TH (n = 320) and PH (n = 381) cohorts or in extended Korean cohorts combined with the TCGA cohort, respectively. *One TCGA sample was from a patient who underwent liver transplantation and was tumor-free and therefore ignored. **c-f** Contingency tables of, respectively, tumor relapse, metastasis, T stage, or Edmondson-Steiner (ES) grade by pretreatment in extended Korean TH cohorts. ES grade information is missing for a sample from one patient with pretreatment.

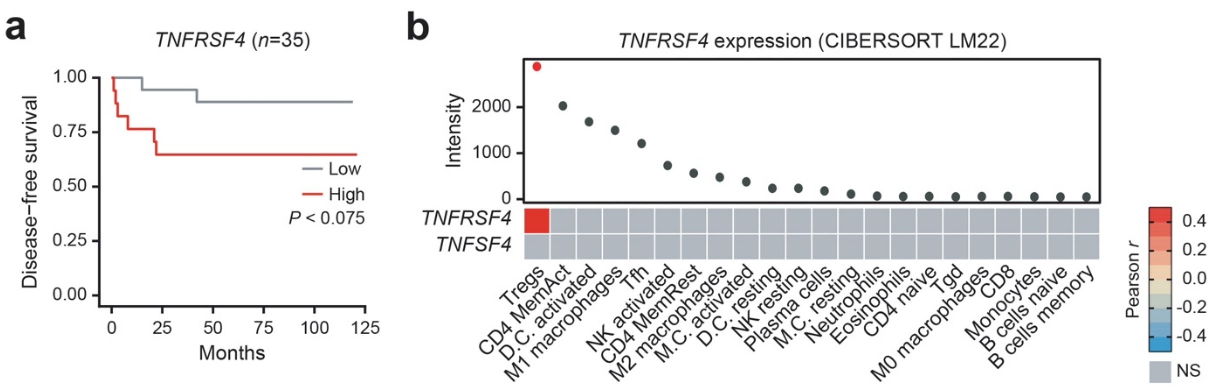

Supplementary Fig. 8. Association of *TNFRSF4* expression with Treg infiltration in the total hepatectomy group. a Kaplan-Meier DFS analysis of patients stratified by *TNFRSF4* expression in the TH group. **b** Expression of *TNFRSF4* in the CIBERSORT LM22 reference matrix (top) and correlations between *TNFRSF4* or *TNFSF4* expression with fractions of different cell types (bottom). Only the Treg fraction was correlated with the expression of *TNFRSF4*. Cells were sorted by *TNFRSF4* expression.

SUPPLEMENTARY TABLES

Supplementary Table 1. Korean HCC cohort

Sample ID	Paired sample[a]	Operational method	Pathology[b]
N001	No	NA	Nontumor
N002	No	NA	Nontumor
N003	Paired_17	Partial hepatectomy	Nontumor
N004	No	NA	Nontumor
N005	No	NA	Nontumor
N006	No	NA	Nontumor
N007	No	NA	Nontumor
N011	No	NA	Nontumor
N012	No	NA	Nontumor
N013	No	NA	Nontumor
N014	No	NA	Nontumor
N015	No	NA	Nontumor
N016	No	NA	Nontumor
N017	No	NA	Nontumor
N018	No	NA	Nontumor
FL001	Paired_4	Partial hepatectomy	Fibrosis low grade
FL002	Paired_21	Total hepatectomy	Fibrosis low grade
FL003	Paired_6	Partial hepatectomy	Fibrosis low grade
FL004	Paired_7	Partial hepatectomy	Fibrosis low grade
FL007	Paired_18	Partial hepatectomy	Fibrosis low grade
FL008	No	NA	Fibrosis low grade
FL010	Paired_9	Partial hepatectomy	Fibrosis low grade
FL012	Paired_19	Partial hepatectomy	Fibrosis low grade
FL013	No	NA	Fibrosis low grade
FL014	Paired_25	Partial hepatectomy	Fibrosis low grade
FH002	Paired_3	Total hepatectomy	Fibrosis high grade
FH005	Paired_2	Partial hepatectomy	Fibrosis high grade
FH007	Paired_15	Total hepatectomy	Fibrosis high grade
FH008	Paired_1	Total hepatectomy	Fibrosis high grade
FH009	No	NA	Fibrosis high grade
FH010	No	NA	Fibrosis high grade
FH014	No	NA	Fibrosis high grade
FH016	Paired_22	Partial hepatectomy	Fibrosis high grade
FH017	Paired_23	Partial hepatectomy	Fibrosis high grade
FH018	Paired_24	Partial hepatectomy	Fibrosis high grade
CS002	Paired_20	Partial hepatectomy	Cirrhosis
CS010	No	NA	Cirrhosis
CS016	No	NA	Cirrhosis
CS017	No	NA	Cirrhosis
CS019	Paired_8	Total hepatectomy	Cirrhosis
CS022	No	NA	Cirrhosis
CS028	Paired_12	Total hepatectomy	Cirrhosis
CS029	Paired_13	NA	Cirrhosis
CS038	No	NA	Cirrhosis

CS039	Paired_16	Total hepatectomy	Cirrhosis
DL002	No	NA	Dysplastic nodule low grade
DL011	No	NA	Dysplastic nodule low grade
DL016	Paired_5	Total hepatectomy	Dysplastic nodule low grade
DL019	No	NA	Dysplastic nodule low grade
DL020	No	NA	Dysplastic nodule low grade
DL021	No	NA	Dysplastic nodule low grade
DL023	No	NA	Dysplastic nodule low grade
DL024	No	NA	Dysplastic nodule low grade
DL025	Paired_13	NA	Dysplastic nodule low grade
DL026	Paired_14	Total hepatectomy	Dysplastic nodule low grade
DH003	No	NA	Dysplastic nodule high grade
DH004	No	NA	Dysplastic nodule high grade
DH008	No	NA	Dysplastic nodule high grade
DH009	Paired_11	Total hepatectomy	Dysplastic nodule high grade
DH010	No	NA	Dysplastic nodule high grade
DH011	No	NA	Dysplastic nodule high grade
DH013	No	Total hepatectomy	Dysplastic nodule high grade
TG1-003	No	Total hepatectomy	ES1
TG1-004	Paired_4	Partial hepatectomy	ES1
TG1-005	No	Total hepatectomy	ES1
TG1-007	Paired_5	Total hepatectomy	ES1
TG1-009	No	Total hepatectomy	ES1
TG1-012	Paired_15	Total hepatectomy	ES1
TG1-013	No	Total hepatectomy	ES1
TG1-017	Paired_14	Total hepatectomy	ES1
TG1-021	No	Total hepatectomy	ES1
TG1-023	No	Total hepatectomy	ES1
TG1-025	No	Partial hepatectomy	ES1
TG2-001	No	Total hepatectomy	ES2
TG2-002	No	Total hepatectomy	ES2
TG2-004	No	Total hepatectomy	ES2
TG2-005	Paired_10	Total hepatectomy	ES2
TG2-006	No	Partial hepatectomy	ES2
TG2-008	No	Total hepatectomy	ES2
TG2-010	No	Total hepatectomy	ES2
TG2-012	No	Total hepatectomy	ES2
TG2-013	No	Total hepatectomy	ES2
TG2-014	Paired_12	Total hepatectomy	ES2
TG2-019	No	Total hepatectomy	ES2
TG2-022	Paired_11	Total hepatectomy	ES2
TG2-023	Paired_16	Total hepatectomy	ES2
TG2-025	Paired_17	Partial hepatectomy	ES2
TG2-026	Paired_6	Partial hepatectomy	ES2
TG2-028	No	Partial hepatectomy	ES2
TG2-029	Paired_18	Partial hepatectomy	ES2
TG2-030	No	Total hepatectomy	ES2
TG2-032	Paired_20	Partial hepatectomy	ES2
TG2-033	No	Total hepatectomy	ES2

TG2-034	Paired_22	Partial hepatectomy	ES2
TG2-035	Paired_21	Total hepatectomy	ES2
TG2-036	Paired_23	Partial hepatectomy	ES2
TG2-037	No	Partial hepatectomy	ES2
TG2-038	Paired_9	Partial hepatectomy	ES2
TG3-001	No	Total hepatectomy	ES3
TG3-002	No	Total hepatectomy	ES3
TG3-003	Paired_10	Total hepatectomy	ES3
TG3-004	Paired_8	Total hepatectomy	ES3
TG3-005	No	Partial hepatectomy	ES3
TG3-006	No	Total hepatectomy	ES3
TG3-007	No	Total hepatectomy	ES3
TG3-008	No	Total hepatectomy	ES3
TG3-009	No	Total hepatectomy	ES3
TG3-010	No	Total hepatectomy	ES3
TG3-012	Paired_3	Total hepatectomy	ES3
TG3-013	Paired_7	Partial hepatectomy	ES3
TG3-015	Paired_1	Total hepatectomy	ES3
TG3-016	No	Partial hepatectomy	ES3
TG3-019	Paired_2	Partial hepatectomy	ES3
TG3-020	No	Total hepatectomy	ES3
TG3-021	Paired_19	Partial hepatectomy	ES3
TG3-022	No	Partial hepatectomy	ES3
TG3-023	Paired_25	Partial hepatectomy	ES3
TG3-024	Paired_24	Partial hepatectomy	ES3
HPC-001	Paired_8	Total hepatectomy	Mixed HCC-CCA
HPC-002	No	Partial hepatectomy	Mixed HCC-CCA
HPC-003	No	Partial hepatectomy	Mixed HCC-CCA
HPC-004	No	NA	Mixed HCC-CCA
HPC-005	No	Partial hepatectomy	Mixed HCC-CCA
HPC-006	No	Total hepatectomy	Mixed HCC-CCA

a;normal/nontumor and tumor pairs

b;ES (Edmonson-Steiner grade), HCC (hepatocellular carcinoma), CCA (cholangiocarcinoma)

Supplementary Table 2. Korean HCC cohort clinical metadata

Sample	Op	Pathology	Risk factor	Progression	Tumor stage	TNM N	TNM M	Age	Sex	Maximum size (cm)	Activity (METAVIR)	Fibrosis (METAVIR)	Ishak score	Child-Pugh	Weight (kg)	Height (cm)	Family history	AFP (ng/ml)	Vital status	OS (month)	Disease-free	DFS (month)
DH013	TH	DN high	HBV	None	Stage II	N0	M0	55	M	1.8	NA	Stage4	6	A	62	155	Yes	31.8	Alive	46	Yes	46
TG1-003	TH	ES1	HBV	None	Stage II	N0	M0	66	M	1.7	NA	Stage4	6	B	NA	NA	NA	23	Alive	102	Yes	102
TG1-005	TH	ES1	HBV	None	Stage II	N0	M0	41	M	3.5	A2	Stage4	6	B	67	170	NA	42	Alive	119	Yes	119
TG1-007	TH	ES1	HCV	None	Stage II	N0	M0	60	M	1.5	NA	Stage4	6	C	75	162	NA	10	Alive	91	Yes	91
TG1-009	TH	ES1	HBV	None	Stage I	N0	M0	53	M	0.5	NA	Stage4	6	C	63	163	Yes	12	Alive	84	Yes	84
TG1-012	TH	ES1	HBV	None	Stage II	N0	M0	43	F	2.9	NA	Stage4	6	B	59	157	No	55.4	Alive	69	Yes	69
TG1-013	TH	ES1	HBV	None	Stage I	N0	M0	52	F	1.5	NA	Stage4	6	C	56	152	Yes	5.1	Alive	68	Yes	68
TG1-017	TH	ES1	HBV	None	Stage II	Nx	Mx	58	M	1.5	NA	NA	NA	A	76	170	NA	10	Alive	71	Yes	71
TG1-021	TH	ES1	HBV	None	Stage II	N0	M0	60	M	3.1	NA	Stage4	6	B	54	177	Yes	8	Alive	101	Yes	101
TG1-023	TH	ES1	HBV	None	Stage IIIA	N0	M0	53	M	5.4	NA	Stage4	6	B	66	173	No	16.2	Alive	45	Yes	45
TG2-001	TH	ES2	HBV	None	Stage II	N0	M0	39	M	1	A2	Stage4	6	B	NA	NA	NA	0	Alive	108	Yes	108
TG2-002	TH	ES2	HBV	None	Stage I	N0	M0	50	F	6	A2	Stage4	6	C	58	155	Yes	1460	Alive	99	Yes	99
TG2-004	TH	ES2	HBV	Local recur	Stage II	N0	M0	48	M	3	NA	Stage4	6	B	77	177	Yes	436	Alive	92	No	8
TG2-005	TH	ES2	HBV	None	Stage II	N0	M0	49	M	4.8	NA	Stage4	6	C	65	172	NA	52	Alive	96	Yes	96
TG2-008	TH	ES2	HBV	None	Stage I	N0	M0	58	M	0.5	NA	NA	NA	A	68	173	Yes	5	Alive	89	Yes	89
TG2-010	TH	ES2	HBV	None	Stage II	N0	M0	58	M	1.5	NA	Stage4	6	C	60	165	No	10	Alive	93	Yes	93
TG2-012	TH	ES2	HBV	None	Stage I	Nx	Mx	42	M	2	NA	Stage4	6	A	74	172	Yes	6	Alive	92	Yes	92
TG2-013	TH	ES2	HBV	Distant meta	Stage IIIC	N0	M0	58	M	4.3	NA	Stage4	6	A	63	165	Yes	15500	Alive	80	No	2
TG2-014	TH	ES2	HBV	None	Stage I	N0	M0	36	M	2	NA	Stage4	6	A	72	168	Yes	10	Alive	79	Yes	79
TG2-019	TH	ES2	HBV	None	Stage II	N0	M0	40	M	1.9	NA	Stage4	6	A	82	177	Yes	5	Alive	82	Yes	82
TG2-022	TH	ES2	HBV	Local recur	Stage II	N0	M0	56	M	4.8	NA	Stage4	6	A	62	163	Yes	172	Dead	13	No	3
TG2-023	TH	ES2	HBV	None	Stage IIIA	N0	M0	56	M	8	NA	Stage4	6	A	58	170	No	18.9	Alive	61	Yes	61
TG2-030	TH	ES2	HBV	None	Stage II	N0	M0	51	M	3	NA	Stage4	6	A	86	174	No	45.2	Alive	46	Yes	46
TG2-033	TH	ES2	HCV	Local recur	Stage IIIA	N0	M0	61	M	6	NA	Stage4	6	C	83	179	No	1570	Alive	22	No	1
TG2-035	TH	ES2	HCV	None	Stage II	N0	M0	62	F	2.8	NA	Stage4	6	B	53	156	No	893	Alive	98	Yes	98
TG3-001	TH	ES3	HBV	Recur&meta	Stage II	N0	M0	62	M	3	NA	Stage4	6	C	61	171	Yes	1980	Dead	22	No	22
TG3-002	TH	ES3	HBV	None	Stage II	N0	M0	38	M	3.2	A1	Stage4	6	B	NA	NA	NA	5	Alive	116	Yes	116
TG3-004	TH	ES3	HBV	None	Stage II	N0	M0	57	M	2.5	NA	Stage4	6	C	88	170	NA	172	Alive	88	Yes	88
TG3-006	TH	ES3	HBV	None	Stage I	N0	M0	60	M	2	NA	Stage4	6	B	66	167	Yes	5	Alive	121	Yes	121
TG3-007	TH	ES3	HBV	None	Stage II	N0	M0	57	M	4	NA	Stage4	6	C	75	164	No	61	Alive	80	Yes	80
TG3-009	TH	ES3	HBV	None	Stage I	N0	M0	52	F	2	NA	Stage4	6	B	45	158	NA	356	Alive	75	Yes	75
TG3-010	TH	ES3	HBV	Local recur	Stage I	N0	M0	65	M	3	NA	Stage4	6	C	72	158	No	6	Dead	26	No	15
TG3-012	TH	ES3	HBV	Recur&meta	Stage IVB	N0	M1	51	M	12	A2	Stage3	5	C	59	163	Yes	6	Dead	84	No	42
TG3-015	TH	ES3	HBV	None	Stage II	N0	M0	54	M	1.8	NA	Stage4	6	A	73	160	Yes	5.6	Alive	58	Yes	58
TG3-020	TH	ES3	HCV	Recur&meta	Stage IIIB	N0	M0	44	M	5.2	NA	Stage4	6	B	82	167	No	200400	Alive	47	No	21
HPC-002	PH	*Mixed	NASH	None	Stage I	N0	M0	55	M	5.2	A1	Stage3	5	A	81	173	No	1524	Alive	47	Yes	47
TG1-004	PH	ES1	nonBnonC	None	Stage I	N0	M0	64	M	3.1	A1	Stage0	0	A	NA	NA	NA	0	Alive	98	Yes	98
TG2-006	PH	ES2	nonBnonC	Local recur	Stage IIIB	N0	M0	52	M	18	A2	Stage4	6	A	70	169	NA	0	Dead	2	No	2
TG2-025	PH	ES2	Alcoholic	Local recur	Stage I	N0	M0	64	M	10.5	A1	Stage0	0	A	73	167	No	5.4	Alive	58	No	12
TG2-026	PH	ES2	HBV	Local recur	Stage I	N0	M0	57	M	1.8	A1	Stage2	3,4	A	69	166	No	26.1	Alive	50	No	31
TG2-028	PH	ES2	HBV	Local recur	Stage IIIB	N0	M0	47	M	12.5	A1	Stage3	5	A	57	172	No	2554	Alive	13	No	4
TG2-029	PH	ES2	nonBnonC	Distant meta	Stage IIIA	N0	M0	78	M	10	A1	Stage2	3,4	A	57	163	No	112	Alive	105	No	3
TG2-032	PH	ES2	HBV	Local recur	Stage IVB	N0	M1	57	M	4.2	A1	Stage3	5	A	66	167	No	52.1	Alive	85	No	6
TG2-034	PH	ES2	nonBnonC	Local recur	Stage IVB	N0	M1	70	M	22	A1	Stage3	5	A	70	164	No	6650	Alive	101	No	7
TG2-036	PH	ES2	nonBnonC	None	Stage II	N0	M0	53	M	9	A2	Stage3	5	A	60	167	No	6.3	Alive	92	Yes	92
TG2-037	PH	ES2	nonBnonC	None	Stage IIIA	Nx	Mx	70	M	7.5	A0	Stage0	0	A	57	158	Yes	2.7	Alive	86	Yes	86
TG2-038	PH	ES2	HBV	Distant meta	Stage II	N0	M0	48	M	5	A1	Stage2	3,4	A	67	174	Yes	23.9	Alive	83	No	25
TG3-005	PH	ES3	HCV	Local recur	Stage I	N0	M0	75	F	5	NA	Stage4	6	NA	58.9	154.8	Yes	177	Dead	35	No	14
TG3-013	PH	ES3	HBV	None	Stage II	N0	M0	50	M	2.5	A1	Stage2	3,4	A	70	164	No	93.8	Alive	58	Yes	58
TG3-016	PH	ES3	HBV	Local recur	Stage II	N0	M0	49	M	5.5	A1	Stage1	1,2	A	68	162	No	21	Alive	8	No	7
TG3-019	PH	ES3	NASH	None	Stage II	N0	M0	66	M	2.2	A2	Stage3	5	A	84	177	Yes	3.5	Alive	92	Yes	92
TG3-021	PH	ES3	HBV	Recur&meta	Stage I	N0	M0	49	F	7.2	A0	Stage2	3,4	A	61	166	Yes	49.1	Alive	44	No	12
TG3-023	PH	ES3	HCV	Local recur	Stage IIIA	N0	M0	70	F	8.2	A1	Stage2	3,4	A	56	150	Yes	176200	Alive	88	No	1
TG3-024	PH	ES3	HBV	Local recur	Stage I	N0	M0	65	M	7.8	A1	Stage3	5	A	66	155	Yes	3.5	Alive	37	No	37

*Mixed: mixed hepatocellular carcinoma and cholangiocarcinoma

Supplementary Table 3. ESTIMATE scores

Sample ID	Stromal score	Immune score	ESTIMATE score	Purity
N001	592.21	1202.81	1795.01	0.65
N002	-205.07	607.28	402.21	0.79
N003	61.52	998.57	1060.09	0.72
N004	1082.09	1291.17	2373.25	0.58
N005	511.84	1433.94	1945.78	0.63
N006	536.7	1308.29	1844.99	0.64
N007	183.07	1158.09	1341.16	0.7
N011	177.06	733.64	910.71	0.74
N012	726.9	1350.8	2077.69	0.61
N013	229.23	1127.71	1356.94	0.69
N014	1042.06	2230.22	3272.27	0.47
N015	151.95	1066.96	1218.91	0.71
N016	134.23	593.02	727.24	0.76
N017	97.24	1205.55	1302.79	0.7
N018	354.87	1276.2	1631.08	0.66
FL001	229.1	1366.23	1595.33	0.67
FL002	371.4	1444.07	1815.48	0.64
FL003	442.34	1891.41	2333.75	0.58
FL004	347.54	1356.68	1704.22	0.66
FL007	471.18	1577.56	2048.74	0.62
FL008	222.82	1118.93	1341.75	0.7
FL010	372.74	1633	2005.74	0.62
FL012	424.86	1411.8	1836.66	0.64
FL013	445.6	1955.05	2400.65	0.58
FL014	553.48	1939.68	2493.16	0.56
FH002	567.29	972.29	1539.58	0.67
FH005	364.04	1554.2	1918.24	0.63
FH007	810.17	1851.89	2662.06	0.54
FH008	566.03	1341.39	1907.42	0.63
FH009	461.68	1426.48	1888.16	0.64
FH010	765.65	1881.96	2647.61	0.55
FH014	932.48	2212.7	3145.18	0.48
FH016	1229.31	2111.05	3340.36	0.46
FH017	1024.31	1436.06	2460.37	0.57
FH018	268.86	1225.96	1494.83	0.68
CS002	664.49	1732.75	2397.24	0.58
CS010	672.49	1763.98	2436.47	0.57
CS016	1052.48	2078.69	3131.17	0.48
CS017	391.53	1111.82	1503.35	0.68
CS019	480.44	1371.75	1852.18	0.64
CS022	1270.7	2133.11	3403.81	0.45
CS028	-449.78	208.93	-240.86	0.84
CS029	782.63	1763.55	2546.18	0.56
CS038	635.1	1358.15	1993.25	0.62
CS039	855.55	1880.63	2736.18	0.53
DL002	407.75	1355.93	1763.68	0.65

DL011	207.21	464.2	671.4	0.76
DL016	469.2	1449.04	1918.24	0.63
DL019	920.14	1819.68	2739.82	0.53
DL020	169.7	764.98	934.68	0.74
DL021	-739.53	508.78	-230.76	0.84
DL023	-102.32	810.28	707.96	0.76
DL024	-342.05	864.35	522.3	0.78
DL025	-322.16	67.74	-254.42	0.84
DL026	661.83	1360.09	2021.92	0.62
DH003	96.41	494.06	590.47	0.77
DH004	515.68	1204.11	1719.79	0.65
DH008	163.07	595.01	758.08	0.75
DH009	84.49	721.02	805.52	0.75
DH010	533.12	873.5	1406.61	0.69
DH011	53.43	625.91	679.34	0.76
DH013	292.37	1144.49	1436.86	0.69
TG1-003	-341.98	399.96	57.98	0.82
TG1-004	-722.89	503.06	-219.83	0.84
TG1-005	574.95	1264.89	1839.84	0.64
TG1-007	347.45	994.67	1342.12	0.7
TG1-009	290.04	1394.63	1684.67	0.66
TG1-012	647.9	1581.11	2229.01	0.6
TG1-013	539.79	1678.16	2217.95	0.6
TG1-017	213.4	716.76	930.17	0.74
TG1-021	754	1635.36	2389.36	0.58
TG1-023	16.75	669.18	685.92	0.76
TG2-001	191.58	17.31	208.89	0.8
TG2-002	156.23	585.5	741.73	0.76
TG2-004	-605.43	-101.63	-707.06	0.88
TG2-005	1185.68	1657.69	2843.37	0.52
TG2-006	865.01	1272.58	2137.58	0.61
TG2-008	269.73	746.44	1016.17	0.73
TG2-010	241.84	892.43	1134.27	0.72
TG2-012	-252.18	658.12	405.93	0.79
TG2-013	1120.24	558.25	1678.49	0.66
TG2-014	349.27	645.59	994.86	0.73
TG2-019	926.71	1749.31	2676.02	0.54
TG2-022	-1216.21	50.72	-1165.49	0.91
TG2-023	-1384.03	-885.46	-2269.49	0.96
TG2-025	-967.9	245.61	-722.29	0.88
TG2-026	271	1834.35	2105.35	0.61
TG2-028	-564	-186.04	-750.04	0.88
TG2-029	-940.27	-644.46	-1584.73	0.93
TG2-030	-995.65	-370.61	-1366.26	0.92
TG2-032	-769.97	250.96	-519.01	0.86
TG2-033	1946.55	1856.79	3803.34	0.4
TG2-034	-900.96	-434.89	-1335.85	0.92
TG2-035	235.83	628.42	864.25	0.74
TG2-036	-942.28	-306.79	-1249.07	0.91
TG2-037	-1548.41	-587.83	-2136.24	0.96

TG2-038	-24.47	132.56	108.08	0.81
TG3-001	-115.62	329.46	213.84	0.8
TG3-002	-193.29	719.89	526.61	0.78
TG3-003	-774.24	507.35	-266.89	0.84
TG3-004	241.28	928.57	1169.85	0.71
TG3-005	-851.76	-208.63	-1060.39	0.9
TG3-006	925.12	1730.35	2655.47	0.54
TG3-007	-698.85	-53.12	-751.97	0.88
TG3-008	539.21	1806.63	2345.83	0.58
TG3-009	41.4	558.38	599.77	0.77
TG3-010	-1316.33	-465.14	-1781.46	0.94
TG3-012	-1420.45	-993.25	-2413.7	0.97
TG3-013	-467.19	46.23	-420.97	0.86
TG3-015	552.01	1140.67	1692.67	0.66
TG3-016	-848.06	-277.04	-1125.1	0.9
TG3-019	-291.97	1.96	-290.01	0.85
TG3-020	301.37	866.51	1167.87	0.71
TG3-021	-679.56	262.31	-417.25	0.86
TG3-023	-739.5	-167.37	-906.88	0.89
TG3-024	-924.12	-213.09	-1137.22	0.91
HPC-001	-373.72	-208.14	-581.86	0.87
HPC-002	-640.94	-578.15	-1219.08	0.91
HPC-003	-574.23	-55.74	-629.97	0.87
HPC-004	682.76	731.37	1414.13	0.69
HPC-005	978.68	2240.1	3218.77	0.47
HPC-006	-417.75	-295.08	-712.84	0.88

Supplementary Table 4. CIBERSORT scores

Absolute	B naive	B memory	Plasma cells	CD8	CD4 naive	CD4 Mem Rest	CD4 Mem Act	Tfh	Treg	Tgd	NK resting	NK activated	Mono.	Mp M0	Mp M1	Mp M2	DC resting	DC activated	MC resting	MC activated	Eosinophils	Neutrophils	*P*-value	Pearson Correlation	Absolute score
N001	0.004	0	0.021	0	0	0.17	0	0.02	0	0	0.057	0	0.074	0	0.002	0.13	0	0	0.017	0	0.0054	0.021	0.24	0.051	0.53
N002	0.014	0	0.021	0	0	0.12	0	0	0	0.004	0.04	0.0027	0.035	0.02	0.012	0.099	0	0	0.023	0	3.00E-04	0.0091	0.48	0.012	0.4
N003	0.003	0	0.027	0	0	0.2	0	0	0	0.028	0.016	0	0.085	0	0.021	0.096	0	0	0.042	0	0	0.036	0.3	0.037	0.55
N004	0.019	0	0.053	0.024	0	0.18	0.013	0	0	0	0	0.0074	0.095	0.015	0.025	0.23	0	0	0	0.0026	0	0.012	0.22	0.057	0.68
N005	0.024	0	0.12	0.017	0	0.25	0	0	0	0.062	0	0.01	0.024	0.019	0.038	0.13	0.047	0	0.0098	0	0	0.014	0.098	0.11	0.76
N006	0.018	0	0.03	0.019	0	0.12	0	0	0.001	0.03	0	0.022	0.03	0	0.034	0.16	0	0	0.026	0	0	0.019	0.26	0.043	0.51
N007	0	0	0.029	0	0	0.13	0	0	0	0.032	0	0.024	0.08	0	0.031	0.13	0	0	0.034	0	0	0.031	0.16	0.077	0.53
N011	0.006	0	0.013	0	0	0.16	0	0	0	0	0.033	0	0.056	0.015	0.031	0.095	0.0051	0	0.026	0	0	0.021	0.33	0.032	0.46
N012	0.027	0	0.014	0.006	0	0.11	0	0.007	0	0.011	0	0.024	0.12	0.018	0.021	0.12	0	0	0	0.18	0.013	0.011	0.11	0.1	0.67
N013	0.026	0	0.007	0	0	0.08	0	0	0	0.028	0	0.018	0.036	0	0.021	0.2	0.0027	0	0	0.039	0.007	0.073	0.098	0.11	0.54
N014	0	0	0.0025	0.034	0	0.16	0	0	0	0.081	0	0.02	0.052	0.016	0.072	0.29	0.015	0	0.041	0	0.0013	0.0084	0.022	0.2	0.79
N015	0	0.0025	0.016	0	0	0.11	0	0.014	0	0.045	0	0.038	0.038	0	0.021	0.16	0	0	0	0.043	0.0013	0.046	0.18	0.068	0.53
N016	0	0.0019	0.02	0	0	0.13	0	0.012	0	0.03	0	0.012	0.055	0	0.007	0.1	0	0	0.03	0	0	0.029	0.34	0.03	0.43
N017	0	0	0.014	0	0	0.18	0.012	0	0	0.079	0	0.0077	0.083	0	0.02	0.24	0	0	0.048	0	0.0013	0.042	0.11	0.1	0.73
N018	0.017	0	0.046	0	0	0.17	8.00E-04	0	0	0.057	0.0098	2.00E-04	0.067	0	0.029	0.13	0	0	0.061	0	0.017	0.028	0.22	0.056	0.64
FL001	0.002	0	0.016	0.04	0	0.12	0.02	0	0	0.02	0.032	0	0.13	0.024	0.054	0.23	0.016	0	0	0.019	0.034	0.03	0.12	0.094	0.8
FL002	0.026	0	0.046	0.008	0	0.091	0	0	0	0.02	0	0.039	0.096	0.029	0.031	0.13	0.023	0	0	0.12	0	0.076	0.058	0.15	0.74
FL003	0.027	0	0.061	0.11	0	0.29	0	0.015	0	0.009	0	0.032	0.074	0	0.078	0.17	0.043	0	0	0.012	0.011	0.015	0.08	0.13	0.94
FL004	0.032	0	0.025	0.091	0	0.13	0	0.004	0	0.022	0.012	0	0.036	0	0.069	0.19	0.067	0	0.023	0	0.0067	0.02	0.14	0.083	0.73
FL007	0.007	0	0.019	0.052	0	0.19	0	0	0	0.012	0.024	0.022	0.086	0.002	0.016	0.23	0.022	0	0.05	0	4.00E-04	0.033	0.12	0.099	0.77
FL008	0.012	0	0.018	0	0	0.13	0	0	0	0.035	0	0.018	0.076	0	0.033	0.21	0	0	0	0.072	0.0013	0.017	0.19	0.064	0.62
FL010	0.018	0	0.059	0.066	0	0.31	0	0.022	0	1.00E-	5.00E-04	0.02	0.026	0.019	0.1	0.19	0.027	0	0.018	0	0	0.024	0.08	0.12	0.9
FL012	0.006	0	0.008	0	0	0.21	0	0	0	0.027	0.011	0.015	0.057	0	0.041	0.25	0.028	0	0.014	0	0.004	0.026	0.096	0.11	0.69
FL013	0.035	0	0.015	0.021	0	0.23	0.0024	0	0	0.057	0	0.028	0.074	0.032	0.11	0.22	0.0095	0	0.064	0	0.0094	0	0.036	0.17	0.9
FL014	0.056	0	0.036	0.034	0	0.24	0	0.001	0	0.066	0	0.016	0.12	0	0.12	0.25	0	0	0.036	0	0.0015	0.015	0.07	0.14	0.99
FH002	0.008	0	0.033	0	0	0.2	0	6.00E-	0	0	0.016	0.013	0.1	0	0.026	0.16	0	0	0	0.036	0.0053	0.017	0.27	0.04	0.61
FH005	0.031	0	0.032	0	0	0.21	7.00E-04	0	0	9.00E-	0.032	0.0059	0.19	0.022	0.037	0.16	0	0.0019	0.0073	0	0.004	0.043	0.16	0.08	0.78
FH007	0.084	0	0.12	0.051	0	0.24	0.015	0	0	0.003	0.0075	0	0.12	0.033	0.094	0.14	0.014	0	0	0.046	0.0094	0.011	0.03	0.18	0.98
FH008	0.044	0	0.084	0.058	0	0.27	0	0	0	0.023	0	0.023	0.054	0.002	0.048	0.13	0.011	3.00E-04	0.031	0	0.0055	0.004	0.17	0.071	0.79
FH009	0.05	0	0.026	0.019	0	0.15	0.0013	0	0	0.028	0.02	0	0.048	0.032	0.095	0.21	0.023	0	0.013	0	0.012	0.014	0.12	0.095	0.74
FH010	0.016	0	0.03	0.1	0	0.23	0	0	0	0.007	0	0.025	0.099	0	0.094	0.15	0.11	0	0.052	0	0	0.0043	0.062	0.14	0.93
FH014	0.005	0	0.1	0	0	0.26	0.043	0	0	0.028	0	0.0081	0.15	0.024	0.047	0.28	0.032	0	0.093	0	0	0.12	0.02	0.2	1.2
FH016	0.053	0	0.19	0	0	0.35	0.027	0	0	0.062	0	0.034	0.13	0	0.024	0.24	0	0	0	0.0038	0.0044	0.052	0.028	0.18	1.2
FH017	0.023	0	0.045	0.021	0	0.19	0	0	0	0.006	0	0.016	0.068	0.021	0.05	0.2	0.053	0	0.033	0	0.0036	0.0024	0.08	0.12	0.74
FH018	0.024	0	0.053	0.005	0	0.16	0	0	0	0.046	0	0.018	0.15	0	0.009	0.16	0	0	0.013	0	0	0.067	0.15	0.082	0.7
CS002	0.029	0	0.14	0.16	0	0.14	0	0.02	0	0.012	0	0.02	0.042	0	0.1	0.23	0.019	0	0.011	0	0	0.0051	0.06	0.14	0.92
CS010	0.03	0	0.08	0.11	0	0.35	0.019	0	0	0.001	2.00E-04	0.0081	0.074	0.037	0.059	0.21	0.047	0	0.025	0	0.016	0.019	0.08	0.12	1.1
CS016	0.044	0	0.057	0.096	0	0.42	0.0061	0	0	0	0.0012	0.019	0.092	0.006	0.023	0.13	0.069	0	0.017	0.0042	0.018	0.0073	0.006	0.25	1
CS017	0.025	0	0.045	0.025	0	0.061	0	4.00E-	0	0.019	0	0.022	0.083	0	0.012	0.24	0	0	0.071	0	0	0.01	0.2	0.06	0.61
CS019	0.008	0	0.028	0.045	0	0.28	0	0	0	0.009	0	0.02	0.056	0.025	0.11	0.17	0.017	0	0.011	0	0.0031	0.015	0.11	0.1	0.8
CS022	0.025	0	0.044	0.053	0	0.3	0.0018	0	0	0.014	0	0.039	0.1	0.062	0.15	0.12	0.031	0	0.11	0	0.014	0	0.004	0.27	1.1
CS028	0.017	0	5.00E-04	5.00E-	0	0.063	0	0	0.002	0.022	0	0.031	0.021	0.021	0.036	0.077	0.043	0	0.019	0	0	0.0024	0.67	-0.01	0.35
CS029	0.028	0	0.077	0.067	0	0.17	0	0.008	0	0.031	0	0.052	0.16	0.035	0.1	0.1	0.046	0	0.019	0	0.01	0.035	0.016	0.21	0.94
CS038	0.043	0	0.044	0	0	0.29	0.023	0	0	0.02	0.013	0	0.055	0.032	0.055	0.18	0.013	0	0.042	0	0.009	0.014	0.13	0.087	0.83
CS039	0.047	0	0.098	0.029	0	0.22	0.011	0	0	0.035	0	0.026	0.058	0.027	0.11	0.26	0	0	0.055	0	0	0.0085	0.036	0.17	0.99
DL002	0.1	0	0.036	0.028	0	0.28	0.026	0	0	0.028	0	0.031	0.031	0.027	0.093	0.19	0.013	0	0.029	0	0.014	0.028	0.13	0.09	0.95
DL011	0.011	0	0.002	0	0	0.15	0	0	0	0.02	0	0.03	0.041	0.026	0.078	0.1	0	0	0.0084	0	0	0.058	0.12	0.095	0.53
DL016	0.017	0	0.11	0.07	0	0.21	0.0029	0	0	0.008	0.0016	0	0.028	0.01	0.089	0.15	0.057	0	0.028	0	0	1.00E-04	0.074	0.13	0.78
DL019	0.023	0	0.094	0.027	0	0.26	0	0	0	0.011	0	0.022	0.076	0.031	0.15	0.082	0.012	0	0.065	0	0	0	0.016	0.21	0.85
DL020	0.011	0	0.026	0.014	0	0.097	0	0	0	0	0	0.039	0.062	0.036	0.033	0.18	0.0016	0	0.059	0	0	0.014	0.28	0.04	0.58
DL021	0.014	0	0.0066	0	0	0.16	0	0.03	0.015	0	0	0.03	0.037	0.1	0.03	0.13	0.041	0	0.016	0	0	0.0029	0.31	0.035	0.61
DL023	0.018	0	0.0024	0	0	0.12	0	0	6.00E-	0.003	0.023	0.024	0.032	0.035	0.064	0.15	0.0081	0	0.057	0	0	0.019	0.19	0.064	0.55
DL024	0.008	0	0.046	0.033	0	0.099	0	0.019	0.008	0.006	0	0.045	0.042	0.014	0.085	0.1	0.0047	0	0.081	0	0	0.012	0.042	0.16	0.61
DL025	0.009	0	0.0088	0	0	0.1	0	0	0.005	0	0	0.012	0.039	0.002	0.037	0.096	0.032	0	0.038	0	0	0	0.84	-0.024	0.38
DL026	0.025	0	0.031	0.001	0	0.2	0	0	0	0	0.027	0.0078	0.017	0.051	0.026	0.31	0.01	0	0.023	0	0	0.02	0.13	0.087	0.75
DH003	0	0	0.0097	0.025	0	0.059	0	0	0.007	0	0.0079	0.0041	0.029	0.017	0.014	0.12	0.0069	0	0.04	0	0.013	0.023	0.27	0.04	0.38
DH004	0.033	0	0.027	0	0	0.15	0	0	0	0.037	0	0.015	0.082	0.027	0.07	0.23	0	0	0.018	0	0.0018	0.038	0.14	0.086	0.72
DH008	0.001	4.00E-04	0.0059	0	0	0.09	0	0	0.007	0	0.029	0.0021	0.036	0.031	0.005	0.098	0	0	0.024	0	0.0036	0.0049	0.31	0.034	0.34
DH009	0.002	0	0.017	0	0	0.14	0	0	9.00E-	0.012	0	0	0.016	0	0.046	0.16	0.0039	0	0.031	0	0	0.0062	0.19	0.067	0.43
DH010	0.026	0	0.0044	0	0	0.12	0	0.01	0	0	0	0.014	0.042	0.011	0.076	0.12	0	0	0	0.033	0	0.073	0.08	0.12	0.53
DH011	0.023	0	0.001	0	0	0.081	0	0	0	0.023	0	0.018	0.021	0	0.009	0.13	0	0	0.026	0	0	0.007	0.95	-0.033	0.34
DH013	0.036	0	0.0034	0	0	0.15	0.0057	0.002	0	0.053	0.0035	0	0.0053	0.071	0.041	0.13	0.0069	0	0.011	0	0	0.0042	0.17	0.074	0.52
TG1-003	0.017	0	0.012	0	0	0.083	0	0.013	0	0.02	0	0.011	0.047	0.017	0.023	0.15	0	0	0	0.0086	0	0.012	0.48	0.013	0.41
TG1-004	0.02	0	0	0	0	0.14	0.0058	0	0	0.017	0	0	0.018	0	0.038	0.046	0.01	0	0.0044	0	0	0	0.19	0.065	0.3
TG1-005	0	0	0.0053	0	0	0.097	9.00E-04	0	0.003	0.009	0.0028	0	0.0046	0.13	0.022	0.18	0.0025	0	0.032	0	0	0	0.016	0.21	0.49
TG1-007	0.005	0	0.03	0.025	0	0.078	0	0.001	0	0.043	0	0.015	0.037	0.02	0.076	0.16	0.051	0	0.025	0	0.0015	0	0.096	0.11	0.56
TG1-009	0.016	0	0.016	9.00E-	0	0.15	0	0	0	0.068	0	0.033	0.037	0.009	0.056	0.12	0.0057	0	0.061	0	0.0014	0.0042	0.08	0.13	0.57
TG1-012	0.023	0	0.026	0	0	0.21	0.005	0	0	0.058	0	0	0.064	0.012	0.059	0.12	0.036	0	0.016	0	0	0.0027	0.11	0.1	0.63
TG1-013	0.034	0	0.082	0.059	0	0.19	0.0065	0	0.004	0.008	0	0.037	0.089	0.009	0.091	0.091	0.0016	0	0.081	0	0.0015	0	0.024	0.19	0.78
TG1-017	0.006	0	0.0047	0	0	0.13	0.006	0	4.00E-	0	0.041	0	0.018	0.018	0.018	0.13	0.0065	0	0.034	0	0	0.046	0.33	0.033	0.46
TG1-021	0.017	0	0.083	0.068	0	0.26	0	0	0	0.006	0	0.036	0.081	0.062	0.041	0.074	0	0	0.019	0	0.004	0.0069	0.052	0.16	0.76
TG1-023	0.004	0	0.006	0	0	0.1	0	0.005	0	0	0.016	0.013	0.046	0	0.007	0.14	0	0	0.031	0	0	0.0022	0.64	-0.0053	0.38
TG2-001	0.006	0	0.016	0	0	0.097	0	0.008	0	0	0	0.0093	0.01	0.01	0.008	0.097	0.0054	0.015	0.0076	0	0	0.0093	0.97	-0.037	0.3
TG2-002	0.023	0	0.014	0	0	0.18	0	0	0	0	0.0073	0.0082	0.024	0.035	0.007	0.067	0.0041	0	0.027	0	0	1.00E-04	0.4	0.022	0.39
TG2-004	0.024	0	0.005	0	0	0.079	0	0	0.011	0	0.0016	0.0061	0.0081	0.039	0.005	0.11	0.004	0	0.0099	0	0.0018	0.0038	0.58	-0.0013	0.31
TG2-005	0.044	0	0.093	0.029	0	0.23	0.0099	0	0	0.025	0	0.0071	0.081	0.023	0.039	0.21	0	0	0.061	0	0	0.022	0.052	0.16	0.88
TG2-006	0.055	0	0.004	0.028	0	0.22	0.035	0	0	0.043	0	0	0.098	0.023	0.077	0.23	0	0	0.043	0	0	0.0063	0.12	0.095	0.86
TG2-008	0.009	0	0.0036	0	0	0.13	0	0	0	0.013	0.015	0.0031	0.057	0.008	0.026	0.14	0.0015	0	0.0095	0	0	0	0.33	0.032	0.42
TG2-010	0.007	0	0.03	0	0	0.18	0	0.011	0.001	0.011	0.012	0.002	0.025	0.026	0.064	0.089	0.013	0	0.018	0	0.0021	0.012	0.26	0.043	0.5
TG2-012	0.003	0	0.0024	0.029	0	0.17	0	0	0	0	0.0038	0.01	0.053	0.013	0.033	0.046	0.035	0	0.035	0	0.002	0	0.34	0.031	0.43
TG2-013	0.008	0	0.021	0	0	0.12	0	0	0.001	0	0.0048	0	5.00E-04	0.064	0.009	0.11	0	0	0.0081	0	0.0012	0	0.08	0.12	0.35
TG2-014	0.016	0	0.008	0	0	0.15	6.00E-04	0	0	0.011	0.006	0	0.019	0.015	0.016	0.097	0.026	0	0.0022	0	0	0.0052	0.51	0.0087	0.37
TG2-019	0.013	0	0.01	0	0	0.28	0.026	0	0	0.034	0	0	0.027	0.04	0.025	0.19	0.072	0.0082	0.019	0	0.018	0	0.024	0.19	0.77
TG2-022	0.007	0	0.024	0	0	0.1	0.038	0	0.004	0	0.0088	0	0.016	0.025	0.036	0.04	1.00E-04	0	0	0	0	0	0.34	0.031	0.3
TG2-023	0.008	0	0.021	0	0	0.028	0	0	0.004	0.002	0.0074	0	0.005	0.003	0.014	0.031	0	0	0	3.00E-04	0	0	0.79	-0.02	0.13
TG2-025	0.01	0	5.00E-04	0	0	0.065	0	0.003	0	0	0.0072	0.0013	0	0.1	0.017	0.031	0	0	0.015	0	0	0	0.038	0.16	0.25
TG2-026	0.014	0	0.021	0.044	0	0.25	0.0085	0	0	0.094	0	0.014	0	0.012	0.078	0.072	0.012	0	0.029	0	0	0	0.024	0.19	0.65
TG2-028	0.036	0	0	0	0	0.089	0.0099	0	0	0	0.014	0	0.022	0	0.016	0.1	0.017	0	0.0016	0	0	0	0.7	-0.012	0.31
TG2-029	0.015	0	0	0	0	0.06	0	0.016	0	0	0	0.0053	0.0048	0	0.02	0.049	0	0.0046	0.018	0	0	0	1	-0.046	0.19
TG2-030	0.003	0	0	0.002	0	0.056	0	0	0	0	0.0083	0	0.018	0.009	0.009	0.048	0.0051	0	0	0.0068	0.006	2.00E-04	0.89	-0.028	0.17
TG2-032	0.001	0	0	0	0	0.1	0	0	0	0.004	0.004	0.005	0.027	0.019	0.033	0.092	0.11	0	0.031	0	0.003	0	0.13	0.088	0.43
TG2-033	0.004	0	0.032	0.002	0	0.16	0.0072	0	0	0	0.011	0.0036	0.17	0.024	0.015	0.26	0	0	0.045	0	0	0.093	0.004	0.29	0.82
TG2-034	0.002	0	0.0039	0	0.009	0.044	0	0.008	0	0	0.026	0.014	0.032	0.013	0.003	0.067	0	0	0.019	0	0	0.016	0.34	0.031	0.26
TG2-035	0.017	0	0.019	0	0	0.18	0.0077	0	0	0.009	0	0.0065	0.021	0.051	0.044	0.13	0.014	0	0.036	0	0.0016	6.00E-04	0.12	0.097	0.54
TG2-036	0.034	0	0.0081	0.008	0	0.042	7.00E-04	0	0.007	0	0.017	0.0029	0.034	0.001	0.038	0.048	0	0	0.018	0	0	0	0.42	0.019	0.26

TG2-037	0	0	0	0	0	0.055	0	0.003	0.03	0	0.019	0	0.019	0.036	0.008	0	0	0	0.017	0	0	0	0.34	0.031	0.19
TG2-038	0.018	0	0.0018	0.031	0	0.096	0.015	0.001	0.005	0	0	0	0.014	0.035	0.008	0.026	6.00E-04	0	0.023	0	0	0.021	0.79	-0.019	0.3
TG3-001	0.017	0	0.0064	0	0	0.075	0.0013	0.004	0.005	0	0.0031	0	0.011	0.011	0.039	0.06	2.00E-04	0	0.021	0	0	0	0.066	0.14	0.25
TG3-002	0.025	0	0	0	0	0.12	0.0057	0	0	0.012	0.0099	0	0.0027	0.02	0.011	0.089	0.04	0	0.0025	0	0.0045	0	0.63	-0.0046	0.34
TG3-003	0.014	0	0.022	0.053	0	0.089	0.0022	0	0	0	0.023	0.0087	0.1	0.004	0.06	0.05	4.00E-04	0	0.043	0	0	0.0069	0.4	0.022	0.48
TG3-004	0.016	0	0.022	0	0	0.17	0.023	0	0	0.013	0	0.0052	0.017	0.045	0.046	0.13	0.042	0	0.03	0	0	0	0.18	0.068	0.56
TG3-005	0.001	0	0.0038	0.044	0	0.062	0	0.014	0	0	0.006	0.015	0.04	0.016	0.027	0.048	0.0046	0	0.058	0	0	0.0029	0.65	-0.0068	0.34
TG3-006	0.065	0	0	0.004	0	0.35	0	0	0	0	0	0.011	0.082	0.011	0.06	0.057	0.089	0	0.028	0	0	0	0.008	0.23	0.76
TG3-007	0.023	0	0.0085	0	0	0.12	0	0	0	0	0.0058	0.0027	0.018	0	0.011	0.078	0.013	0	0.016	0	8.00E-04	0.0098	0.97	-0.037	0.31
TG3-008	0.005	0	0.024	0	0	0.17	0.022	0.016	0.006	0	0	0.014	0.0055	0.079	0.078	0.12	7.00E-04	0	0.073	0	0.0025	0.013	0.004	0.27	0.64
TG3-009	0.006	0	0.0032	0	0	0.12	6.00E-04	0	0	0	0.017	0	0.021	0	0.054	0.077	0	0	0.0086	0	0	0.0067	0.55	0.0032	0.32
TG3-010	0.011	0	0	0.014	0	0.039	0.002	0.009	0.008	0	0.0072	0	0.0052	0.051	0.008	0.028	0	0	0	0	0	0	0.85	-0.024	0.18
TG3-012	0.029	0	0.0054	0	0	0.07	0	0	0.007	7.00E-	0	0.0023	3.00E-04	0	0.002	0.034	0	0.0013	0.0023	0	0.0035	0	0.99	-0.044	0.16
TG3-013	0.012	0	0.0084	0	0	0.09	0	0.001	0	0	0.0057	0	0.012	0.021	0.017	0.079	0.021	0	0.011	0	0	0	0.84	-0.024	0.28
TG3-015	0.027	0	0.027	0	0	0.2	2.00E-04	0	0	0.031	0	0.013	0.033	0	0.04	0.098	4.00E-04	0	0.014	0	0.0027	0	0.35	0.029	0.49
TG3-016	0.003	0	0	0	0	0.066	0	0	0.002	0.006	0	0.0059	0.012	0.037	0.014	0.089	0.0088	0	0.031	0	0	0	0.71	-0.013	0.28
TG3-019	0.021	0	0.0014	0	0	0.087	0.0055	0.005	0	0.001	0.0065	0	0.0069	0.036	0.007	0.053	0	0	0	0.0013	0	8.00E-04	0.65	-0.0068	0.23
TG3-020	0.003	0	0.022	0.051	0	0.055	0	0	9.00E-	0.011	0	0.036	0.045	0.073	0.02	0.089	0.0097	0	0.074	0	0	0.017	0.1	0.11	0.51
TG3-021	0.004	0	9.00E-04	5.00E-	0	0.08	2.00E-04	9.00E-	0	0	0	0.0033	0.014	0.001	0.006	0.075	0.0011	0	0.028	0	0	0.0023	0.51	0.0089	0.22
TG3-023	0.029	0	5.00E-04	0.002	0	0.066	0	0.003	0	0.005	0	0.025	0.029	0.045	0.023	0.076	0.0035	0	0.028	0	0	0.0087	0.37	0.026	0.34
TG3-024	0	0	2.00E-04	0.001	0	0.052	0	0	0	0	0	0.015	0.024	0.061	0.007	0.014	0	0	0.037	0	0.0081	0.027	0.098	0.11	0.25
HPC-001	0.001	0	0.0062	0.005	0	0.077	0	0	0	0	0	0	0.017	0	0.007	0.037	2.00E-04	0	0.03	0	0	0	0.99	-0.043	0.18
HPC-002	0.005	0	0	0	0	0.09	0	0	0	0	0	4.00E-04	0	0	0.009	0.052	0.0089	0	0.0047	0	0	0	0.94	-0.032	0.17
HPC-003	0.024	0	0.0073	0	0	0.064	0	0	0	0	0.011	0.0054	0.019	0	0.025	0.088	4.00E-04	0	0.018	0	0	0	0.96	-0.035	0.26
HPC-004	0.013	0	0.028	0.001	0	0.12	0.013	0	0	0.036	0	0	0	0.08	0.042	0.11	1.00E-04	0	0.012	0	0	0	0.25	0.044	0.46
HPC-005	0.029	0	0.016	0.027	0	0.053	0.054	0.004	0	0.025	0.0042	0	0.0092	0.15	0.17	0.27	0.0053	0.023	0.067	0	0.015	0.062	0.002	0.33	0.97
HPC-006	0.002	0	0.0013	0.013	0	0.075	0	0	0.007	0	0.031	0	0.026	0.029	0.003	0.015	0	0	0.021	0	0.0016	0.0066	0.98	-0.039	0.23

Relative	B naive	B memory	Plasma cells	CD8	CD4 naive	CD4 Mem Rest	CD4 Mem Act	Tfh	Treg	Tgd	NK resting	NK activated	Mono.	Mp M0	Mp M1	Mp M2	DC resting	DC activated	MC resting	MC activated	Eosinophils	Neutrophils	*P*-value	Pearson Correlation	RMSE
N001	0.008	0	0.041	0	0	0.33	0	0.038	0	0	0.11	0	0.14	0	0.005	0.25	0	0	0.033	0	0.01	0.04	0.24	0.051	1.1
N002	0.036	0	0.053	0	0	0.3	0	0	0	0.01	0.1	0.0052	0.084	0.051	0.031	0.25	0	0	0.056	0	0.0021	0.022	0.46	0.012	1.1
N003	0.005	0	0.048	0	0	0.36	0	0	0	0.05	0.029	0	0.15	0	0.037	0.17	0	0	0.076	0	0	0.065	0.3	0.037	1.1
N004	0.028	0	0.078	0.035	0	0.27	0.019	0	0	0	0	0.011	0.14	0.022	0.036	0.34	0	0	0	0.0038	0	0.018	0.22	0.057	1.1
N005	0.031	0	0.16	0.022	0	0.33	0	0	0	0.081	0	0.013	0.031	0.025	0.05	0.17	0.062	0	0.013	0	0	0.018	0.092	0.11	1
N006	0.036	0	0.059	0.037	0	0.24	0	0	0.002	0.058	0	0.043	0.058	0	0.067	0.31	0	0	0.052	0	0	0.037	0.27	0.043	1.1
N007	0	0	0.056	0	0	0.25	0	0	0	0.061	0	0.046	0.15	0	0.059	0.25	0	0	0.065	0	0	0.059	0.17	0.077	1.1
N011	0.013	0	0.027	0	0	0.35	0	0	0	0	0.071	0	0.12	0.033	0.066	0.2	0.011	0	0.055	0	0	0.045	0.34	0.032	1.1
N012	0.041	0	0.021	0.009	0	0.16	0	0.011	0	0.016	0	0.035	0.18	0.026	0.032	0.17	0	0	0	0.27	0.019	0.016	0.11	0.1	1.1
N013	0.047	0	0.013	0	0	0.15	0	0	0	0.053	0	0.032	0.066	0	0.039	0.38	0.0049	0	0	0.072	0.013	0.14	0.092	0.11	1.1
N014	0	0	0.006	0.058	0	0.17	0.0053	0	0	0.12	0	0	0.078	0.011	0.11	0.39	0.013	0	0.036	0	0.0048	0.0038	0.026	0.2	1
N015	0	0.0047	0.03	0	0	0.2	0	0.026	0	0.084	0	0.072	0.072	0	0.039	0.3	0	0	0	0.08	0.0024	0.087	0.19	0.068	1.1
N016	0	0.0044	0.046	0	0	0.31	0	0.029	0	0.07	0	0.027	0.13	0	0.017	0.24	0	0	0.069	0	0	0.068	0.34	0.03	1.1
N017	0	0	0.019	0	0	0.25	0.016	0	0	0.11	0	0.01	0.11	0	0.027	0.33	0	0	0.065	0	0.0018	0.058	0.11	0.1	1.1
N018	0.026	0	0.072	0	0	0.27	0.0012	0	0	0.089	0.015	3.00E-04	0.11	0	0.045	0.21	0	0	0.096	0	0.026	0.044	0.22	0.056	1.1
FL001	0.003	0	0.02	0.05	0	0.16	0.024	0	0	0.025	0.04	0	0.17	0.03	0.068	0.29	0.02	0	0	0.024	0.043	0.037	0.11	0.094	1.1
FL002	0.035	0	0.062	0.011	0	0.12	0	0	0	0.028	0	0.052	0.13	0.039	0.042	0.18	0.031	0	0	0.17	0	0.1	0.046	0.15	1
FL003	0.029	0	0.065	0.12	0	0.3	0	0.016	0	0.012	0	0.033	0.078	0	0.083	0.18	0.045	0	0	0.013	0.012	0.016	0.062	0.13	1
FL004	0.043	0	0.035	0.12	0	0.18	0	0.006	0	0.028	0.018	0	0.05	0	0.095	0.27	0.091	0	0.031	0	0.011	0.028	0.15	0.083	1.1
FL007	0.009	0	0.024	0.068	0	0.25	0	0	0	0.016	0.031	0.029	0.11	0.002	0.021	0.3	0.029	0	0.064	0	5.00E-04	0.043	0.11	0.099	1
FL008	0.023	0	0.033	0.005	0	0.2	0	0	0	0.045	0	0.029	0.13	0	0.055	0.33	0	0	0	0.12	0.0027	0.025	0.2	0.065	1.1
FL010	0.02	0	0.066	0.073	0	0.35	0	0.024	0	1.00E	5.00E-04	0.022	0.029	0.021	0.12	0.21	0.03	0	0.02	0	0	0.027	0.08	0.12	1
FL012	0.009	0	0.012	0	0	0.3	0	0	0	0.039	0.016	0.022	0.082	0	0.06	0.36	0.041	0	0.02	0	0.0058	0.037	0.088	0.11	1
FL013	0.039	0	0.017	0.023	0	0.25	0.0027	0	0	0.063	0	0.031	0.083	0.035	0.12	0.24	0.011	0	0.071	0	0.01	0	0.034	0.17	1
FL014	0.055	0	0.036	0.039	0	0.24	0	0.001	0	0.064	0	0.015	0.12	0	0.12	0.26	0	0	0.036	0	0.0014	0.015	0.05	0.14	1
FH002	0.013	0	0.053	0	0	0.33	0	0.001	0	0	0.026	0.022	0.16	0	0.042	0.26	0	0	0	0.059	0.0086	0.027	0.27	0.04	1.1
FH005	0.039	0	0.04	0	0	0.27	0.0014	0	0	0.003	0.041	0.0063	0.25	0.03	0.047	0.2	0	0.0022	0.0085	0	0.005	0.055	0.15	0.08	1.1
FH007	0.085	0	0.12	0.053	0	0.24	0.015	0	0	0.003	0.0077	0	0.12	0.033	0.096	0.14	0.014	0	0	0.047	0.0096	0.012	0.032	0.18	1
FH008	0.055	0	0.11	0.074	0	0.35	0	0	0	0.029	0	0.029	0.069	0.003	0.061	0.16	0.014	4.00E-04	0.04	0	0.0069	0.0051	0.18	0.071	1.1
FH009	0.07	0	0.035	0.034	0	0.2	0.0019	0	0	0.038	0.024	0.0013	0.065	0.043	0.13	0.28	0.03	0	0.019	0	0.016	0.019	0.11	0.094	1.1
FH010	0.018	0	0.032	0.11	0	0.25	0	0	0	0.008	0	0.027	0.11	0	0.1	0.17	0.12	0	0.056	0	0	0.0046	0.048	0.14	1
FH014	0.004	0	0.086	0	0	0.22	0.036	0	0	0.024	0	0.0068	0.13	0.02	0.039	0.23	0.027	0	0.079	0	0	0.097	0.026	0.2	1
FH016	0.046	0	0.16	0	0	0.3	0.023	0	0	0.053	0	0.029	0.11	0	0.02	0.21	0	0	0	0.0033	0.0038	0.045	0.032	0.18	1
FH017	0.032	0	0.061	0.029	0	0.26	0	0	0	0.006	0	0.023	0.093	0.029	0.068	0.27	0.071	0	0.046	0	0.0049	0.0033	0.08	0.12	1
FH018	0.034	0	0.075	0.008	0	0.22	0	0	0	0.066	0	0.026	0.22	0	0.013	0.22	0	0	0.019	0	0	0.095	0.15	0.082	1.1
CS002	0.031	0	0.15	0.17	0	0.15	0	0.022	0	0.013	0	0.021	0.046	0	0.11	0.25	0.02	0	0.012	0	0	0.0055	0.048	0.14	1
CS010	0.017	0	0.11	0	0	0.36	0.031	0	0	0.044	0.041	0	0.094	0	0.052	0.16	0.049	0	0.032	0	0	0.015	0.094	0.11	1
CS016	0.043	0	0.056	0.095	0	0.42	0.006	0	0	0	0.0012	0.019	0.091	0.006	0.023	0.12	0.068	0	0.017	0.0042	0.018	0.0072	0.012	0.25	0.99
CS017	0.041	0	0.073	0.041	0	0.1	0	6.00	0	0.03	0	0.036	0.14	0	0.019	0.39	0	0	0.12	0	0	0.017	0.2	0.06	1.1
CS019	0.01	0	0.035	0.056	0	0.34	0	0	0	0.016	0	0.025	0.07	0.032	0.14	0.21	0.018	0	0.015	0	0.0038	0.019	0.11	0.1	1.1
CS022	0.023	0	0.042	0.05	0	0.28	0.0017	0	0	0.014	0	0.037	0.098	0.059	0.14	0.11	0.029	0	0.1	0	0.014	0	0.01	0.27	0.97
CS028	0.047	0	0.0013	0.001	0	0.18	0	0	0.007	0.062	0	0.088	0.059	0.06	0.1	0.22	0.12	0	0.052	0	0	0.0067	0.72	-0.01	1.1
CS029	0.029	0	0.082	0.071	0	0.18	0	0.009	0	0.033	0	0.055	0.17	0.038	0.11	0.11	0.048	0	0.021	0	0.011	0.037	0.024	0.21	1
CS038	0.052	0	0.051	0	0	0.35	0.028	0	0	0.024	0.015	0	0.067	0.035	0.067	0.22	0.02	0	0.05	0	0.011	0.017	0.14	0.086	1
CS039	0.048	0	0.099	0.029	0	0.22	0.011	0	0	0.035	0	0.027	0.059	0.027	0.11	0.26	0	0	0.056	0	0	0.0086	0.032	0.17	1
DL002	0.11	0	0.038	0.03	0	0.3	0.027	0	0	0.03	0	0.032	0.033	0.028	0.098	0.2	0.013	0	0.03	0	0.014	0.03	0.13	0.09	1
DL011	0.021	0	0.0047	0	0	0.29	0	0	0	0.032	0	0.055	0.079	0.049	0.15	0.19	0	0	0.017	0	0	0.11	0.11	0.096	1.1
DL016	0.021	0	0.14	0.09	0	0.27	0.0036	0	0	0.011	0.0022	0	0.036	0.013	0.11	0.19	0.073	0	0.036	0	0	1.00E-04	0.056	0.13	1
DL019	0.027	0	0.11	0.032	0	0.31	0	0	0	0.013	0	0.025	0.089	0.037	0.17	0.096	0.015	0	0.076	0	0	0	0.024	0.21	1
DL020	0.018	0	0.045	0.025	0	0.17	0	0	0	0	0	0.068	0.11	0.062	0.058	0.32	0.0027	0	0.1	0	0	0.024	0.28	0.04	1.1
DL021	0.023	0	0.011	0	0	0.26	0	0.048	0.024	0	0	0.048	0.061	0.17	0.049	0.21	0.066	0	0.027	0	0	0.0046	0.31	0.035	1.1
DL023	0.032	0	0	0	0	0.22	0	0	8.00	0.011	0.047	0.038	0.058	0.062	0.11	0.27	0.015	0	0.097	0	0	0.034	0.2	0.063	1.1
DL024	0.014	0	0.076	0.055	0	0.16	0	0.031	0.014	0.011	0	0.074	0.069	0.022	0.14	0.17	0.0078	0	0.13	0	0	0.02	0.036	0.16	1
DL025	0.027	0	0.023	0	0	0.27	0	0	0.015	0	0	0.026	0.1	0.007	0.099	0.25	0.083	0	0.096	0	0	0	0.84	-0.023	1.1
DL026	0.029	0	0.042	0.002	0	0.26	0	0	0	0	0.037	0.0099	0.023	0.069	0.036	0.41	0.013	0	0.031	0	0	0.027	0.14	0.087	1.1
DH003	0	0	0.025	0.065	0	0.16	0	0	0.02	0	0.021	0.011	0.077	0.046	0.037	0.32	0.018	0	0.1	0	0.035	0.062	0.28	0.04	1.1
DH004	0.049	0	0.037	0	0	0.21	0	0	0	0.05	0	0.022	0.11	0.037	0.096	0.31	0	0	0.025	0	0.0028	0.052	0.14	0.085	1.1
DH008	0.003	0.0012	0.019	0	0	0.28	0	0	0.022	0	0.071	0.015	0.1	0.091	0.015	0.28	0	0	0.072	0	0.01	0.014	0.34	0.032	1.1
DH009	0.005	0	0.038	0	0	0.32	0	0	0.002	0.027	0	0	0.037	0	0.11	0.36	0.0092	0	0.073	0	0	0.014	0.19	0.067	1.1
DH010	0.05	0	0.0084	0	0	0.24	0	0.02	0	0	0	0.026	0.079	0.02	0.14	0.22	0	0	0	0.062	0	0.14	0.072	0.12	1.1
DH011	0.066	0	0.0028	0	0	0.24	0	0	0	0.068	0	0.053	0.059	0	0.027	0.39	0	0	0.077	0	0	0.02	0.95	-0.033	1.1
DH013	0.07	0	0.0066	0	0	0.29	0.011	0.004	0	0.1	0.0066	0	0.01	0.13	0.079	0.24	0.013	0	0.021	0	0	0.008	0.17	0.074	1.1
TG1-003	0.041	0	0.03	0	0	0.2	0	0.032	0	0.049	0	0.026	0.11	0.042	0.056	0.36	0	0	0	0.021	0	0.03	0.45	0.013	1.1
TG1-004	0.066	0	0	0	0	0.48	0.019	0	0	0.055	0	0	0.058	0	0.12	0.15	0.034	0	0.014	0	0	0	0.2	0.065	1.1
TG1-005	0	0	0.011	0	0	0.2	0.0018	0	0.007	0.019	0.0057	0	0.0093	0.26	0.044	0.37	0.0052	0	0.065	0	0	0	0.024	0.21	1
TG1-007	0.009	0	0.053	0.044	0	0.14	0	0.003	0	0.077	0	0.026	0.066	0.036	0.14	0.28	0.09	0	0.044	0	0.0027	0	0.088	0.11	1
TG1-009	0.014	0	0	0.046	0	0.24	0.014	0	0	0.12	0	0	0.05	0.021	0.13	0.34	0.013	0	0.012	0	0	0	0.038	0.16	1
TG1-012	0.009	0	0.013	0	0	0.38	0.0029	0	0	0.052	0	0.016	0.078	0.032	0.095	0.19	0.064	0	0.053	0	0	0.0097	0.09	0.11	1
TG1-013	0.043	0	0.1	0.075	0	0.24	0.0083	0	0.005	0.01	0	0.048	0.11	0.013	0.12	0.12	0.0021	0	0.1	0	0.0019	0	0.03	0.19	1
TG1-017	0.013	0	0.01	0	0	0.28	0.0082	0	0	0	0.08	0.0034	0.04	0.038	0.039	0.3	0.014	0	0.077	0	0	0.1	0.31	0.035	1.1
TG1-021	0.022	0	0.11	0.089	0	0.35	0	0	0	0.008	0	0.048	0.11	0.081	0.054	0.097	0	0	0.026	0	0.0053	0.009	0.038	0.16	1
TG1-023	0.012	0	0.015	0	0	0.28	0	0.015	0	0	0.058	0.02	0.12	0	0.018	0.37	0	0	0.077	0	0	0.0058	0.66	-0.0059	1.1
TG2-001	0.021	0	0.052	0	0	0.32	0	0.031	0	0	0	0.031	0.032	0.033	0.027	0.32	0.021	0.048	0.025	0	0	0.032	0.98	-0.037	1.1
TG2-002	0.058	0	0.037	0	0	0.45	0	0	0	0	0.019	0.021	0.061	0.089	0.019	0.17	0.01	0	0.068	0	0	4.00E-04	0.39	0.022	1.1
TG2-004	0.084	0	0.013	0	0	0.26	0	0	0.036	0	0.0012	0.019	0.026	0.13	0.019	0.35	0.013	0	0.031	0	0.0059	0.012	0.61	-9.00E-04	1.1
TG2-005	0.05	0	0.11	0.033	0	0.27	0.011	0	0	0.028	0	0.0081	0.092	0.026	0.044	0.24	0	0	0.069	0	0	0.025	0.038	0.16	1

TG2-006	0.064	0	0.0047	0.033	0	0.26	0.041	0	0	0.049	0	0	0.11	0.026	0.089	0.26	0	0	0.049	0	0	0.0074	0.11	0.095	1
TG2-008	0.023	0	0.0086	0	0	0.31	0	0	0	0.03	0.037	0.0073	0.14	0.02	0.061	0.34	0.0035	0	0.023	0	0	0	0.33	0.032	1.1
TG2-010	0.014	0	0.059	0	0	0.36	0	0.021	0.003	0.021	0.025	0.0039	0.049	0.052	0.13	0.18	0.025	0	0.035	0	0.0042	0.024	0.27	0.043	1.1
TG2-012	0.008	0	0.0055	0.067	0	0.39	0	0	0	0	0.0088	0.023	0.12	0.029	0.076	0.11	0.082	0	0.08	0	0.0046	0	0.34	0.031	1.1
TG2-013	0.022	0	0.059	0	0	0.34	0	0	0.005	0	0.015	0	0.0011	0.18	0.026	0.32	0	0	0.022	0	0.0032	0	0.074	0.12	1.1
TG2-014	0.039	0	0.022	0	0	0.4	0.0033	0	0	0.024	0.021	0	0.053	0.044	0.042	0.26	0.07	0	0.0063	0	0	0.014	0.49	0.009	1.1
TG2-019	0.017	0	0.013	0	0	0.37	0.034	0	0	0.044	0	0	0.035	0.052	0.032	0.25	0.094	0.011	0.025	0	0.023	0	0.03	0.19	1
TG2-022	0.027	0	0.079	0	0	0.33	0.12	0	0.02	0	0.034	0	0.054	0.085	0.12	0.13	2.00E-04	0	0	0	0	0	0.34	0.03	1.1
TG2-023	0.07	0	0.16	0	0	0.22	0	0	0.031	0.019	0.059	0	0.036	0.033	0.12	0.25	0	0	0	0	0	0	0.8	-0.018	1.1
TG2-025	0.042	0	0.0019	0	0	0.25	0	0.015	0	0	0.028	0.0034	0	0.4	0.07	0.12	0	0	0.059	0	0	0	0.036	0.17	1.1
TG2-026	0.025	0	0.033	0.071	0	0.38	0.013	0	0	0.15	0	0.024	0	0.017	0.12	0.11	0.018	0	0.048	0	0	0	0.03	0.19	1
TG2-028	0.12	0	0	0	0	0.29	0.032	0	0	0	0.045	0	0.071	0	0.051	0.33	0.057	0	0.0052	0	0	0	0.73	-0.012	1.1
TG2-029	0.076	0	0	0	0	0.31	0	0.082	0	0	0	0.028	0.025	0	0.1	0.26	0	0.024	0.093	0	0	0	0.99	-0.046	1.1
TG2-030	0.021	0	0	0.019	0	0.32	0	0	0	0	0.045	0	0.11	0.054	0.057	0.27	0.034	0	0	0.036	0.034	0	0.9	-0.028	1.1
TG2-032	0.003	0	0	0	0	0.24	0	0	0	0.019	0.0056	0.011	0.061	0.043	0.076	0.21	0.26	0	0.072	0	0.0072	0	0.14	0.088	1.1
TG2-033	0.005	0	0.039	0.003	0	0.2	0.0088	0	0	0	0.014	0.0044	0.2	0.029	0.018	0.31	0	0	0.055	0	0	0.11	0.008	0.29	0.97
TG2-034	0.008	0	0.015	0	0.037	0.17	0	0.031	0	0	0.1	0.056	0.13	0.05	0.012	0.26	0	0	0.072	0	0	0.061	0.34	0.031	1.1
TG2-035	0.036	0	0.037	0	0	0.33	0.014	0	0	0.018	0	0.013	0.038	0.094	0.08	0.24	0.027	0	0.066	0	0.0032	9.00E-04	0.11	0.096	1
TG2-036	0.11	0	0.02	0	0	0.27	0	0	0.005	0	0.053	0.029	0.17	0.022	0.14	0.12	0	0	0.055	0	0	0	0.38	0.023	1.1
TG2-037	0	0	0	0	0	0.3	0	0.017	0.16	0	0.099	0	0.098	0.19	0.047	0	0	0	0.093	0	0	0	0.34	0.029	1.1
TG2-038	0.061	0	0.006	0.11	0	0.32	0.051	0.006	0.019	0	0	0	0.049	0.12	0.029	0.087	0.0021	0	0.078	0	0	0.07	0.81	-0.019	1.1
TG3-001	0.065	0	0.026	0	0	0.3	0	0.019	0.022	0	0.0075	0	0.042	0.041	0.16	0.24	0.0011	0	0.083	0	0	0	0.048	0.14	1
TG3-002	0.074	0	0	0	0	0.34	0.017	0	0	0.034	0.03	0	0.0084	0.058	0.033	0.26	0.12	0	0.0072	0	0.013	0	0.65	-0.0046	1.1
TG3-003	0.028	0	0.046	0.11	0	0.19	0.0046	0	0	0	0.048	0.018	0.21	0.009	0.13	0.1	9.00E-04	0	0.09	0	0	0.014	0.39	0.022	1.1
TG3-004	0.032	0	0.039	0	0	0.3	0.041	0	0	0.021	0	0.0079	0.031	0.082	0.085	0.23	0.07	0	0.057	0	0	0	0.19	0.068	1.1
TG3-005	0.004	0	0.011	0.13	0	0.18	0	0.04	0	0	0.017	0.045	0.12	0.046	0.078	0.14	0.013	0	0.17	0	0	0.0085	0.68	-0.0068	1.1
TG3-006	0.086	0	0	0.005	0	0.46	0	0	0	0	2.00E-04	0.014	0.11	0.014	0.081	0.076	0.12	0	0.038	0	0	0	0.018	0.24	0.99
TG3-007	0.076	0	0.028	0	0	0.4	0	0	0	0	0.019	0.0087	0.057	0	0.035	0.25	0.042	0	0.051	0	0.0023	0.031	0.98	-0.037	1.1
TG3-008	0.008	0	0.037	0	0	0.27	0.034	0.024	0.01	0	0	0.022	0.0086	0.12	0.12	0.19	0.0011	0	0.11	0	0.0039	0.021	0.012	0.27	0.98
TG3-009	0.019	0	0.01	0	0	0.39	0.0023	0	0	0.002	0.055	0	0.066	0	0.17	0.24	0	0	0.027	0	0	0.021	0.58	0.0029	1.1
TG3-010	0.058	0	0	0.077	0	0.21	0.011	0.05	0.044	0	0.04	0	0.029	0.28	0.044	0.15	0	0	0	0	0	0	0.86	-0.024	1.1
TG3-012	0.18	0	0.034	0	0	0.44	0	0	0.046	0.004	0	0.015	0.0016	0	0.016	0.21	0	0.0081	0.015	0	0.022	0	0.99	-0.044	1.1
TG3-013	0.041	0	0.03	0	0	0.32	0	0.005	0	0	0.022	0	0.045	0.076	0.062	0.28	0.075	0	0.041	0	0	0	0.86	-0.024	1.1
TG3-015	0.056	0	0.056	0	0	0.42	0.0016	0	0	0.062	0	0.021	0.067	0	0.082	0.21	4.00E-04	0	0.025	0	0.0057	0	0.34	0.03	1.1
TG3-016	0.012	0	0	0	0	0.23	0	0	0.004	0.026	0	0.025	0.043	0.14	0.05	0.32	0.032	0	0.12	0	0	0	0.75	-0.014	1.1
TG3-019	0.088	0	0.0058	0	0	0.37	0.023	0.024	0	0.005	0.028	0	0.029	0.16	0.034	0.23	0	0	0	0.0054	0	0.0033	0.68	-0.0068	1.1
TG3-020	0.006	0	0.043	0.1	0	0.11	0	0	0.001	0.021	0	0.07	0.089	0.14	0.039	0.18	0.019	0	0.15	0	0	0.034	0.098	0.11	1
TG3-021	0.02	0	0.0051	0.004	0	0.37	0.001	0.004	0	0	0	0.015	0.064	0.004	0.03	0.34	0.005	0	0.13	0	0	0.0099	0.5	0.0081	1.1
TG3-023	0.083	0	0.0015	0.006	0	0.19	0	0.009	0	0.015	0	0.074	0.083	0.13	0.066	0.22	0.01	0	0.082	0	0	0.025	0.36	0.026	1.1
TG3-024	0	0	9.00E-04	0.004	0	0.21	0	0	0	0	0	0.061	0.096	0.25	0.031	0.057	0	0	0.15	0	0.033	0.11	0.09	0.11	1.1
HPC-001	0.005	0	0.034	0.03	0	0.42	0	0	0	0	0	0	0.092	0	0.044	0.2	0.0012	0	0.17	0	0	0	0.99	-0.043	1.1
HPC-002	0.03	0	0	0	0	0.53	0	0	0	0	0	0.0024	0	0	0.055	0.3	0.052	0	0.028	0	0	0	0.94	-0.032	1.1
HPC-003	0.09	0	0.028	0	0	0.25	0	0	0	0	0.04	0.021	0.074	0	0.094	0.34	0.0014	0	0.065	0	0	0	0.96	-0.034	1.1
HPC-004	0.029	0	0.061	0.003	0	0.27	0.028	0	0	0.079	0	0	0	0.18	0.093	0.23	3.00E-04	0	0.027	0	0	0	0.26	0.044	1.1
HPC-005	0.029	0	0.017	0.028	0	0.057	0.055	0.005	0	0.025	0.0041	0	0.0095	0.15	0.17	0.27	0.0051	0.025	0.069	0	0.016	0.063	0.004	0.33	0.96
HPC-006	0.008	0	0.0056	0.058	0	0.32	0	0	0.03	0	0.13	0	0.11	0.12	0.016	0.067	0	0	0.091	0	0.0068	0.028	0.98	-0.039	1.1

Supplementary Table 5. Immunohistochemistry cell count

(# of cell per mm^2)	CD3 T cells	CD8 cytotoxic T cells	CD45RO memory T cells	FoxP3 Tregs	MUM1 plasma cells	CD68 macrophages	CD163 macrophages M2	MPO neutrophils	CD45 total leukocytes
**N001**	187	105	166	2	0	845	544	656	429
**N002**	121	120	128	0	0	692	56	20	277
**N003**	295	206	79	1	0	752	340	139	366
**N004**	286	108	214	7	113	538	274	27	171
**N005**	302	246	238	1	28	927	232	124	374
**N006**	408	354	255	1	24	1131	530	65	587
**N007**	191	145	120	0	39	730	383	38	270
**N012**	288	241	118	18	6	1444	879	110	678
**N013**	126	77	62	9	2	795	430	139	233
**N014**	361	38	507	0	15	2128	637	133	805
**N015**	130	102	121	1	2	678	215	59	248
**N016**	174	191	205	0	19	785	251	137	374
**N017**	403	297	313	0	2	1091	516	162	708
**FL001**	241	189	126	2	1	1277	805	116	610
**FL003**	287	270	223	0	51	590	88	12	513
**FL004**	830	620	1289	5	37	753	160	26	397
**FL007**	188	134	207	5	18	577	204	39	216
**FL008**	195	176	211	1	8	930	215	78	446
**FL012**	362	251	423	0	17	583	105	74	307
**FL013**	167	116	161	0	3	909	253	21	243
**FH002**	69	41	47	40	15	849	325	43	272
**FH007**	190	175	341	0	44	631	86	36	237
**FH008**	233	198	195	0	51	458	290	42	278
**FH009**	296	192	163	0	24	813	382	45	286
**FH010**	214	238	198	1	9	966	397	66	379
**FH016**	219	150	386	3	76	885	285	16	198
**CS002**	156	134	143	1	2	581	167	20	191
**CS016**	978	495	854	1	23	479	37	127	574
**CS017**	440	305	304	2	22	440	223	39	204
**CS019**	311	281	195	2	7	703	214	29	336
**CS022**	271	236	172	2	0	637	87	25	328
**CS028**	324	213	124	0	2	519	223	70	580
**CS029**	201	223	243	0	6	315	61	27	247
**CS038**	132	70	61	1	6	597	140	50	172
**CS039**	438	232	218	2	65	1407	513	70	609
**DL002**	62	40	81	1	0	297	190	37	243
**DL011**	19	17	15	0	1	101	17	41	39
**DL016**	672	370	427	0	0	360	183	99	1385
**DL019**	75	59	39	5	0	967	370	29	481
**DL020**	58	38	29	5	0	NA	NA	NA	312
**DL021**	37	53	26	0	0	137	27	22	191
**DL023**	436	180	334	3	3	268	170	62	772
**DL024**	75	66	28	1	1	610	408	57	801
**DL025**	59	50	45	12	5	398	109	22	414
**DL026**	105	30	5	2	1	1415	613	97	1084
**DH003**	32	8	12	0	1	97	6	13	119
**DH004**	139	88	44	1	1	554	432	59	247
**DH008**	241	69	262	14	1	470	187	32	202
**DH009**	148	103	77	1	0	694	248	26	736
**DH010**	110	65	93	0	1	281	42	285	131
**DH011**	232	100	94	1	1	368	154	81	600
**DH013**	153	88	70	0	0	430	368	72	643
**TG1-003**	129	115	44	0	0	919	783	97	320
**TG1-004**	574	209	204	1	1	446	381	47	270
**TG1-005**	211	185	70	5	15	851	242	32	187
**TG1-007**	351	212	233	1	0	135	156	156	599
**TG1-009**	603	484	577	1	3	1313	1140	89	982
**TG1-012**	159	87	114	0	0	433	6	15	707
**TG1-013**	1145	1150	956	2	0	540	323	16	1946
**TG1-017**	499	73	0	1	1	974	591	42	194
**TG1-021**	83	91	70	0	0	1151	220	101	240
**TG1-023**	76	91	32	0	0	486	43	373	73
**TG2-001**	93	46	12	0	0	428	59	5	1743
**TG2-002**	318	174	119	3	1	788	226	15	252
**TG2-004**	102	13	88	0	0	716	33	78	82
**TG2-005**	268	152	91	2	4	505	141	45	341
**TG2-006**	49	34	34	1	0	2	13	28	35
**TG2-008**	922	435	638	0	2	1057	1130	10	583
**TG2-010**	381	188	138	2	0	923	100	241	94
**TG2-013**	66	41	92	1	2	437	277	11	38
**TG2-014**	118	123	124	1	0	11	49	20	178
**TG2-019**	280	49	183	0	0	458	85	19	118
**TG2-022**	257	543	832	6	63	1801	608	558	1119
**TG2-023**	15	6	1	3	0	203	83	7	84
**TG2-025**	207	74	25	8	5	338	67	1	197
**TG2-026**	2764	2727	850	2	5	562	36	16	2629
**TG2-028**	608	159	367	31	2	615	65	8	591
**TG2-029**	52	46	69	2	3	137	2	2	107
**TG2-030**	103	71	44	0	0	252	1	11	127
**TG2-032**	917	269	637	117	62	556	3	9	554
**TG2-033**	42	40	19	0	0	394	81	12	55
**TG2-034**	8	7	2	1	1	145	106	7	39
**TG2-035**	554	192	478	24	6	1864	541	51	460
**TG3-001**	109	116	67	0	-1	965	19	29	160
**TG3-002**	325	437	626	0	1	647	266	16	580
**TG3-003**	485	454	429	0	0	437	51	87	288
**TG3-004**	579	324	451	0	0	1324	840	74	94
**TG3-006**	270	338	140	0	0	198	124	128	313
**TG3-007**	1472	1585	1278	0	0	1323	1667	12	1662
**TG3-008**	649	842	448	0	5	1407	439	349	162

**TG3-009**	991	625	1616	0	0	1213	858	1	225
**TG3-010**	904	809	974	0	0	1318	NA	346	308
**TG3-012**	7	4	6	2	0	115	11	1	44
**TG3-013**	884	96	498	26	60	657	28	1	707
**TG3-015**	815	638	350	2	0	764	323	106	366
**TG3-016**	37	15	25	2	4	319	6	9	73
**TG3-020**	410	222	237	33	8	719	59	11	552
**TG3-021**	314	73	85	14	2	125	141	5	327
**HPC-002**	126	35	105	18	3	176	11	0	35
**HPC-003**	620	494	88	3	2	1051	77	132	790
**HPC-005**	985	411	308	40	42	1478	26	53	473
**HPC-006**	51	59	6	0	0	795	6	52	38
