# Supplementary material for: Preoperative immune landscape predisposes adverse outcomes in hepatocellular carcinoma patients with liver transplantation
Source: NPJ Precis Oncol. 2021 Mar 26;5:27. doi: 10.1038/s41698-021-00167-2 (PMC7997876; doi:10.1038/s41698-021-00167-2)
Supplement: Supplementary file 2 — REPORTING SUMMARY [file 41698_2021_167_MOESM2_ESM.pdf]

nature research

Corresponding author(s): Jin-Wu Nam, Kyoung Bun Lee

Last updated by author(s): Feb 10, 2021

Reporting Summary

Nature Research wishes to improve the reproducibility of the work that we publish. This form provides structure for consistency and transparency in reporting. For further information on Nature Research policies, see our Editorial Policies and the Editorial Policy Checklist.

Statistics

For all statistical analyses, confirm that the following items are present in the figure legend, table legend, main text, or Methods section.

n/a	Confirmed	
☐	☒	The exact sample size (*n*) for each experimental group/condition, given as a discrete number and unit of measurement
☐	☒	A statement on whether measurements were taken from distinct samples or whether the same sample was measured repeatedly
☐	☒	The statistical test(s) used AND whether they are one- or two-sided<br>*Only common tests should be described solely by name; describe more complex techniques in the Methods section.*
☐	☒	A description of all covariates tested
☐	☒	A description of any assumptions or corrections, such as tests of normality and adjustment for multiple comparisons
☐	☒	A full description of the statistical parameters including central tendency (e.g. means) or other basic estimates (e.g. regression coefficient) AND variation (e.g. standard deviation) or associated estimates of uncertainty (e.g. confidence intervals)
☐	☒	For null hypothesis testing, the test statistic (e.g. *F*, *t*, *r*) with confidence intervals, effect sizes, degrees of freedom and *P* value noted<br>*Give P values as exact values whenever suitable.*
☒	☐	For Bayesian analysis, information on the choice of priors and Markov chain Monte Carlo settings
☒	☐	For hierarchical and complex designs, identification of the appropriate level for tests and full reporting of outcomes
☐	☒	Estimates of effect sizes (e.g. Cohen's *d*, Pearson's *r*), indicating how they were calculated

Our web collection on statistics for biologists contains articles on many of the points above.

Software and code

Policy information about availability of computer code

Data collection	Excel, R (3.2.3 & 3.5.3), Python (2.7.5), FastQC (0.11.5), Bowtie (1.0.0), Seqtk (1.0-r31), Sickle (v1.33), STAR (2.5.2b), FeatureCounts (v1.5.1), ESTIMATE (1.0.13), CIBERSORT (1.06), NMF (0.20.6), DAVID Bioinformatics Resources 6.8, Cytoscape (3.5.1 & 3.8.2), and Monocle 2 (v2.10.1)
Data analysis	All RNA-seq data were processed and normalized using Python and R. All the statistical analyses were performed using R. In-house R codes used to produce major figures in this study are available on GitHub (https://github.com/sangho1130/KOR_HCC).

For manuscripts utilizing custom algorithms or software that are central to the research but not yet described in published literature, software must be made available to editors and reviewers. We strongly encourage code deposition in a community repository (e.g. GitHub). See the Nature Research guidelines for submitting code & software for further information.

Data

Policy information about availability of data

All manuscripts must include a data availability statement. This statement should provide the following information, where applicable:

- Accession codes, unique identifiers, or web links for publicly available datasets
- A list of figures that have associated raw data
- A description of any restrictions on data availability

The normalized gene expression and raw data supporting the conclusions of this article are available in the NCBI Gene Expression Omnibus (GEO) database under accession number GSE148355. In-house R codes used to produce major figures in this study are available on GitHub (https://github.com/sangho1130/KOR_HCC).

April 2020

Field-specific reporting

Please select the one below that is the best fit for your research. If you are not sure, read the appropriate sections before making your selection.

☒ Life sciences ☐ Behavioural & social sciences ☐ Ecological, evolutionary & environmental sciences

For a reference copy of the document with all sections, see nature.com/documents/nr-reporting-summary-flat.pdf

Life sciences study design

All studies must disclose on these points even when the disclosure is negative.

Sample size	Total of 62 tumor samples, 47 premalignant, and 15 nontumor samples were collected from our HCC project. For additional public dataset, 418 TCGA LIHC, 447 Japanese HCC (EGAD00001001880), 60 Chinese HCC (GSE77509), and 132 GTEx normal liver samples were processed.
Data exclusions	Two RNA-seq samples TG3-022 and TG1-025 which had low rates of uniquely mapped reads to the human reference genome hg19 using STAR aligner (4.20% and 4.14%, respectively) were excluded in the study.
Replication	Four RNA-seq samples (N013, TG1-017, TG2-035, and HPC-003) were replicated and marked sample's name with "r", otherwise, sequenced once per a patient in with or without paired samples.
Randomization	No randomization was performed. Patients who had surgical resection at Seoul National University Hospital between 2004 and 2009 were subjected for this study.
Blinding	A person who analyzed data (Sang-Ho Yoon) is different from one who prepared tissue RNA-seq samples (Kyoung Bun Lee and Suk Woo Nam). Curation of clinical metadata and immunohistochemistry staining were performed by Kyoung Bun Lee.

Reporting for specific materials, systems and methods

We require information from authors about some types of materials, experimental systems and methods used in many studies. Here, indicate whether each material, system or method listed is relevant to your study. If you are not sure if a list item applies to your research, read the appropriate section before selecting a response.

Materials & experimental systems

n/a	Involved in the study	
☐	☒	Antibodies
☒	☐	Eukaryotic cell lines
☒	☐	Palaeontology and archaeology
☒	☐	Animals and other organisms
☐	☒	Human research participants
☒	☐	Clinical data
☒	☐	Dual use research of concern

Methods

n/a	Involved in the study	
☒	☐	ChIP-seq
☒	☐	Flow cytometry
☒	☐	MRI-based neuroimaging

Antibodies

Antibodies used	CD3 (Ventana; 790-4341; Rabbit monoclonal (2GV6)), CD8 (NOVO; PA0183; Mouse monoclonal (4B11)), CD45Ro (Ventana; 790-2930; Mouse monoclonal (UCHL-1)), Foxp3 (Abcam; ab20034; Mouse monoclonal (236A/E7)), CD68 (DAKO; M0814; Mouse monoclonal (KP1)), CD163 (NOVO; NCL-CD163; Mouse monoclonal (10D6)), MUM1 (DAKO; M7259; Mouse monoclonal (MUM1P)), MPO (DAKO; A0398; Rabbit polypclonal)
Validation	All the antibodies used in this study are previously validated.

Human research participants

Policy information about studies involving human research participants

Population characteristics	Korean HCC patients who had surgical resection at Seoul National University Hospital between 2004 and 2009 were sequenced in pared or un-paired manner. Total of 62 tumor samples were collected. Nontumor samples were collected from patients with metastatic cancer, cholangiocarcinoma, or polycystic liver disease after histological confirmation. One nontumor sample was collected from a metastatic cancer patient who underwent partial hepatectomy, and other 14 nontumor samples were collected from patients with cholangiocarcinoma, or polycystic liver disease after histological confirmation. Additional 47 premalignant lesions were prepared in which 24 samples were paired with tumor samples.
Recruitment	Patients who had surgical resection at Seoul National University Hospital between 2004 and 2009 were subjected for

April 2020

Recruitment	sequencing after receiving written informed consent from the patients according to the Declaration of Helsinki.
Ethics oversight	The Institutional Review Board of Seoul National University Hospital approved this study (H-1501-042-639)

Note that full information on the approval of the study protocol must also be provided in the manuscript.
